# A Yeast BiFC-seq Method for Genome-wide Interactome Mapping

**DOI:** 10.1016/j.gpb.2021.02.008

**Published:** 2021-07-24

**Authors:** Limin Shang, Yuehui Zhang, Yuchen Liu, Chaozhi Jin, Yanzhi Yuan, Chunyan Tian, Ming Ni, Xiaochen Bo, Li Zhang, Dong Li, Fuchu He, Jian Wang

**Affiliations:** 1 State Key Laboratory of Proteomics, Beijing Proteome Research Center, National Center for Protein Sciences (Beijing), Beijing Institute of Lifeomics, Beijing 102206, China; 2 Department of Biotechnology, Beijing Institute of Radiation Medicine, Beijing 100850, China; 3 Department of Rehabilitation Medicine, Nan Lou; Key Laboratory of Wound Repair and Regeneration of PLA, College of Life Sciences, Chinese PLA General Hospital, Beijing 100853, China; 4 School of Basic Medical Sciences, Anhui Medical University, Hefei 230032, China

**Keywords:** Bimolecular fluorescence complementation, Protein–protein interaction, High-throughput, Next-generation sequencing

## Abstract

Genome-wide physical **protein–protein interaction** (PPI) mapping remains a major challenge for current technologies. Here, we reported a high-efficiency BiFC-seq method, yeast-enhanced green fluorescent protein-based **bimolecular fluorescence complementation** (yEGFP-BiFC) coupled with next-generation DNA sequencing, for interactome mapping. We first applied yEGFP-BiFC method to systematically investigate an intraviral network of the Ebola virus. Two-thirds (9/14) of known interactions of EBOV were recaptured, and five novel interactions were discovered. Next, we used the BiFC-seq method to map the interactome of the tumor protein p53. We identified 97 interactors of p53, more than three-quarters of which were novel. Furthermore, in a more complex background, we screened potential interactors by pooling two BiFC libraries together and revealed a network of 229 interactions among 205 proteins. These results show that BiFC-seq is a highly sensitive, rapid, and economical method for genome-wide interactome mapping.

## Introduction

Genome-wide yeast two-hybrid (Y2H) screening and affinity purification coupled with mass spectrometry (AP-MS) have been extensively used for mapping the interactomes of various species [1], [2], [3], [4]. However, only limited coverage is obtained for the interactomes of most organisms [5], [6] because of the low sensitivity, labor-intensiveness, and high cost of these technologies.

The bimolecular fluorescence complementation (BiFC) assay is a powerful tool to investigate binary protein–protein interactions (PPIs). A Venus-based BiFC method has been used in large-scale interactome mapping of telomere signaling and SUMO [7], [8] that specifically detects transient or weak interactions which cannot be obtained by Y2H or AP-MS. However, there is high background fluorescence intensity, which increases the number of artificial interactions [9].

To overcome these limitations, we developed a BiFC-seq method that combines yeast-enhanced green fluorescent protein-based BiFC (yEGFP-BiFC) with next-generation sequencing (NGS), expanding the BiFC application for genome-wide interactome screening. First, we used the yEGFP-BiFC method to depict a small-scale intraviral PPI network of the Ebola virus (EBOV) among its 9 encoded proteins that contained 14 interactions among 6 proteins. Then, we applied BiFC-seq in a high-throughput assay to screen p53 interactors from a universal human library and revealed 97 p53 interactors, with 21 reported in the literature. Finally, we carried out genome-wide interactome screening by pooling two tagged libraries together using BiFC-seq, generating an interaction network consisting of 229 interactions, 12 of which are reported in the BioGrid database.

## Method

### Construction of the yEGFP-BiFC vectors

The yEGFP-BiFC constructs were generated using the pDBLeu and pPC86 vectors (Catalog No. 10835031, Invitrogen, Grand Island, NY) as templates according to standard molecular techniques. Briefly, the coding regions of the DNA activation domain in the pPC86 vector and the DNA binding domain in the pDBLeu vertor were removed, and then the DNA fragments coding for the N- and C-terminal fluorescent proteins, as well as a linker coding for a 15-amino acid spacer sequence (GGGGS)3, were inserted, respectively. The remaining vectors were constructed with these backbone vectors using standard molecular techniques (File S1).

### Construction of the cDNA library

The cDNA library was constructed using Gateway recombination technology. Human universal reference total RNA (Catalog No. 636538, Clontech, Mountain View, CA) was used as a template to synthesize cDNA by reverse transcription. The cDNA was ligated to adaptors with the *att*B1 site. To construct the Gateway Entry vectors, pDONR222 (Catalog No. 54648, Invitrogen) was mixed with the purified cDNA fragments and the BP Clonase II enzyme (Catalog No. 11789100, Invitrogen). The reactions were incubated at 25 °C overnight and then treated with Proteinase K at 37 °C for 10 min for termination. The reaction products were transformed into *E. coli* DH10B competent cells, colonies were grown in LB medium with kanamycin selection, and plasmids were extracted with a Plasmid Mini Kit (Catalog No. 12125, Qiagen, Hilden, Germany). To construct the cDNA library, pDONR222 entry vectors were mixed with the pPC86-YN157-CCDB vectors and the Gateway LR Clonase II enzyme (Catalog No. 11791100, Invitrogen). The reactions were incubated at 25 °C overnight and then treated with Proteinase K at 37 °C for 10 min for termination. The reaction products were transformed into *E. coli* DH10B competent cells, colonies were grown in LB medium with ampicillin selection, and plasmids were extracted and stored at −80 °C.

### yEGFP-BiFC assay

The yeast strain *S. cerevisiae* AH109 (*MAT*a, *trp*1-901, *leu*2-3, 112, *ura*3-52, *his*3-200, *gal*4Δ, *gal*80Δ, *LYS*2:*GAL*1*_UAS_*-*GAL*1*_TATA_*-*HIS*3, *GAL*2*_UAS_*-*GAL*2*_TATA_*-*ADE*2, and *URA*3:*MEL*1*_UAS_*-*MEL*1*_TATA_*-*lacZ*) (Catalog No. 630490, Clontech) was used in the BiFC screening. A small-scale sequential transformation procedure was performed using protocols from the manufacturer. After transformation, the yeast cells were resuspended in 10 ml of liquid SD medium without tryptophan and leucine (SD-2 medium), cultured in a 30 °C shaker for 24 h, and incubated at 4 °C for an additional 48 h for fluorophore maturation. The yeast cells were collected by centrifugation at 7000 r/min for 30 s and resuspended in PBS. Yeast cells with reconstituted yEGFP were excited with a 488-nm laser and collected through a FACSAria III (BD Biosciences, Franklin Lakes, NJ) 530/30 nm bandpass filter. The sorted yeast cells were collected with PBS, spread onto SD-2 plates, and cultured in a 30 °C incubator for 2 days until yeast colonies reached 2–3 mm in diameter. Fluorescence at 488 nm excitation and 519 nm emission was observed under an inverted fluorescence microscope (Olympus, Tokyo, Japan).

### Yeast colony PCR

The yeast colonies were picked and digested with 20 μl of 0.02 N NaOH. After 5 min of boiling, 2 μl of the solution was used as a template to amplify the inserted cDNA with the forward primer 5′-ATGGCTGACAAACAAAGATCTGGT-3′ and the reverse primer 5′- GCGGCCGCACCACTTTGTACAAGAAA-3′. The reaction buffers were mixed and aliquoted with a Biomek FX Laboratory Automation Workstation (Beckman Coulter, Brea, CA) into 384-well PCR plates. PCR was performed with I-5 2× High-Fidelity Master Mix (Catalog No. I5HM-200, MCLAB, TSINGKE Biological Technology) according to the manufacturer’s instructions. For each amplification, 3 μl of the PCR products was mixed and purified using a QIAquick PCR Purification Kit (Catalog No. 28104, Qiagen).

### NGS and BiFC-seq workflow

The purified PCR products were sheared with a Covaris S220 system (Applied Biosystems, Carlsbad, CA). To prepare the libraries, the sheared PCR products were processed using a Nextera XT DNA Sample Preparation Kit according to the manufacturer’s instructions (Illumina, San Diego, CA). All libraries were sequenced at a 2 × 150 bp read length on an Illumina HiSeq 2000 platform. The images generated by the HiSeq 2000 were converted to raw reads using base calling (CASAVA version 1.8). After trimming the adaptor sequences from the raw reads, the reads with ≥ 10% unidentified bases (Ns) and ≥ 50% low-quality bases (PHRED quality scores ≤ 5) were also removed to implement quality control. The filtered reads were mapped to the GRCh38 genome (Human GENCODE version 22) with TopHat [10]. The raw counts of the sequencing reads for each transcript were calculated with the HTSeq Python package [11]. DESeq2 was then used for differential expression analysis between the p53 and control groups [12]. Gene Ontology (GO) analysis was then performed with the clusterProfiler R package (http://bioconductor.org/packages/release/bioc/html/clusterProfiler.html).

### Co-immunoprecipitation assay

HEK293 cells were cultured in Dulbecco’s modified Eagle’s medium (Catalog No. A4192101, ThermoFisher Scientific, San Jose, CA) with 10% (v/v) fetal bovine serum (Catalog No. 16140071, ThermoFisher Scientific). After 48 h of transfection, HEK293 cells with Flag-p53 and Myc-tagged interactors were harvested and lysed in HEPES lysis buffer [0.5% NP-40, 150 mM NaCl, 1% (v/v) Tween 20, 50 mM Tris–HCl pH 7.5, 0.1% 1 M DTT, and a protease inhibitor cocktail]. Co-mmunoprecipitation (Co-IP) was performed using an anti-Myc antibody (Catalog No. 05-724, Sigma-Aldrich, St. Louis, MO) and protein A/G-agarose (Catalog No. 20421, ThermoFisher Scientific) at 4 °C. The lysates and immunoprecipitates were incubated with an anti-Flag antibody (Catalog No. PA1-984B, ThermoFisher Scientific), and then detection was performed with ECL substrate (Catalog No. 32109, ThermoFisher Scientific).

## Results and discussion

### Development of yeast BiFC based on yEGFP

**Figure 1:**
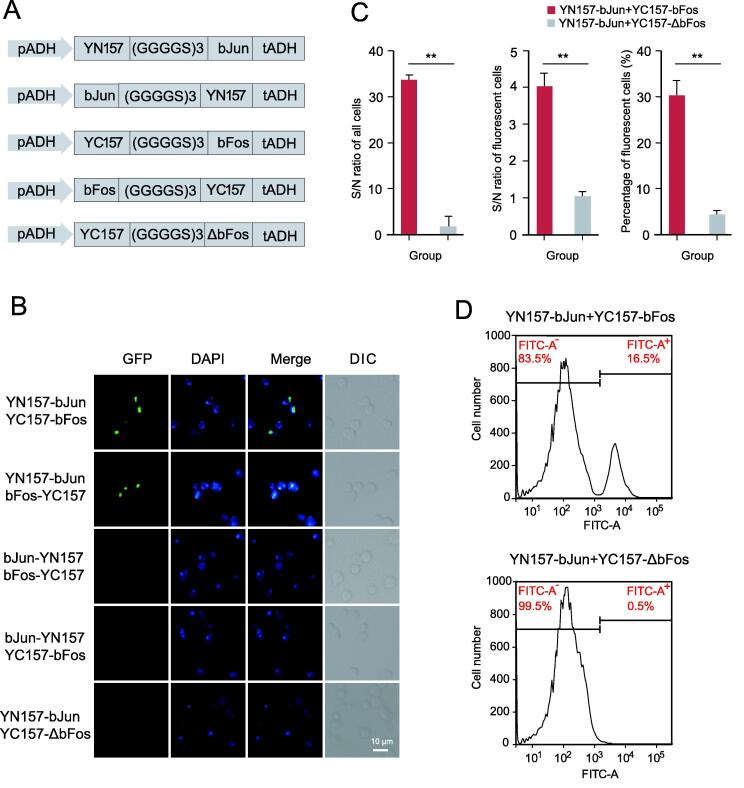
**Development of yEGFP-BiFC** **A.** Diagram of the different fusion patterns of yEGFP split fragments to target proteins. **B.** Representative images showing the yEGFP-BiFC results. The nuclei were labeled with DAPI. Scale bar, 10 μm. **C.** Quantitative results of the fluorescence intensity of the fluorescent yeast cells. For the S/N ratio of all cells, 200 transformed yeast cells from the positive or negative group were randomly selected. The fluorescence intensities were analyzed with ImageJ and summed for each group to calculate the S/N of all cells. For the S/N ratio of fluorescent cells, the fluorescent intensities of 200 fluorescent cells from the positive or negative group were summed for each group to calculate the S/N ratio of fluorescent cells. Data are presented as mean ± SD of three biological replicates. **, *P* value < 0.01 (Student’s *t*-test). **D.** Approximately 20,000 yeast cells from positive and negative groups were analyzed by flow cytometry, and the fluorescence-positive cell ratios were 16.5% and 0.5%, respectively. yEGFP-BiFC, yeast-enhanced green fluorescent protein-based BiFC; S/N, signal-to-noise; DIC, differential interference contrast.

A codon-optimized yEGFP [13] was split into nonfluorescent halves at amino acid 157 to yield the fragments YN157 (amino acids 1–157) and YC157 (amino acids 158–238). Intact yEGFP had a strong fluorescence signal in yeast, whereas split yEGFP fragments produced undetectable fluorescence (Figure S1). To verify that this approach could be used for detecting PPIs, fragments of yEGFP linked by the (GGGGS)3 peptide sequence were fused to the N-termini of bJun (YN157-bJun), bFos (YC157-bFos), and ΔbFos (YC157-ΔbFos) as positive and negative indicators (Figure 1A). Co-transfection of YN157-bJun and YC157-bFos led to the reassembly of yEGFP and strong fluorescence signals. However, the combination of YN157-bJun and YC157-ΔbFos resulted in slight background fluorescence (Figure 1B). The signal-to-noise (S/N) ratio of the yEGFP-BiFC method was 33:1 for all cells and 4:1 for fluorescent cells, indicating that the method could be used for high-sensitivity detection of PPIs. The percentage of fluorescent cells in the positive group was 7-fold higher than that in the negative group under identical transfection conditions (Figure 1C). As expected, the percentage of fluorescence-positive cells was 16.5% as determined by FACS, which was approximately 32-fold higher than the percentage in the negative control group (Figure 1D). The different fusion patterns of interacting proteins with the yEGFP fragments potentially affected the proximity of the split fluorescent protein fragments. Thus, YN157 and YC157 were fused to the C-termini of bJun (bJun-YN157) and bFos (bFos-YC157), respectively (Figure 1A). We found that YN157-bJun combinded with bFos-YC157 aslo produced strong fluorescence signals, wheares bJun-YN157 combinded with YC157-bFos or bFos-YC157 only produced weak background fluorescence (Figure 1B). These results suggest that the yEGFP-based BiFC system is suitable for PPI screening in yeast and that the fusion patterns of yEGFP fragments to targets can potentially affect screening results.

### A comprehensive depiction of Ebola intraviral PPI network

The recent West African epidemic of EBOV disease caused more than 11,000 deaths owing to the high infectivity and lethality of EBOV. Although tremendous efforts have been made to develop EBOV therapeutics, an effective treatment remains elusive. Studies on the pathogenic mechanisms of EBOV have been based mainly on pathogen–host PPI features [14], [15]. However, the intraviral PPI network is still unclear. Therapeutic strategies targeting interactions among internal EBOV proteins can be promising. Thus, we carried out a comprehensive analysis of the interactions of EBOV proteins with each other using yEGFP-BiFC. The EBOV genome encodes nine proteins: nucleoprotein (NP), viral proteins (VP24, VP30, VP35, and VP40), glycoprotein (GP), and polymerase (L), as well as another two isoforms of GP (sGP and GP2) generated through RNA editing [16].

**Figure 2:**
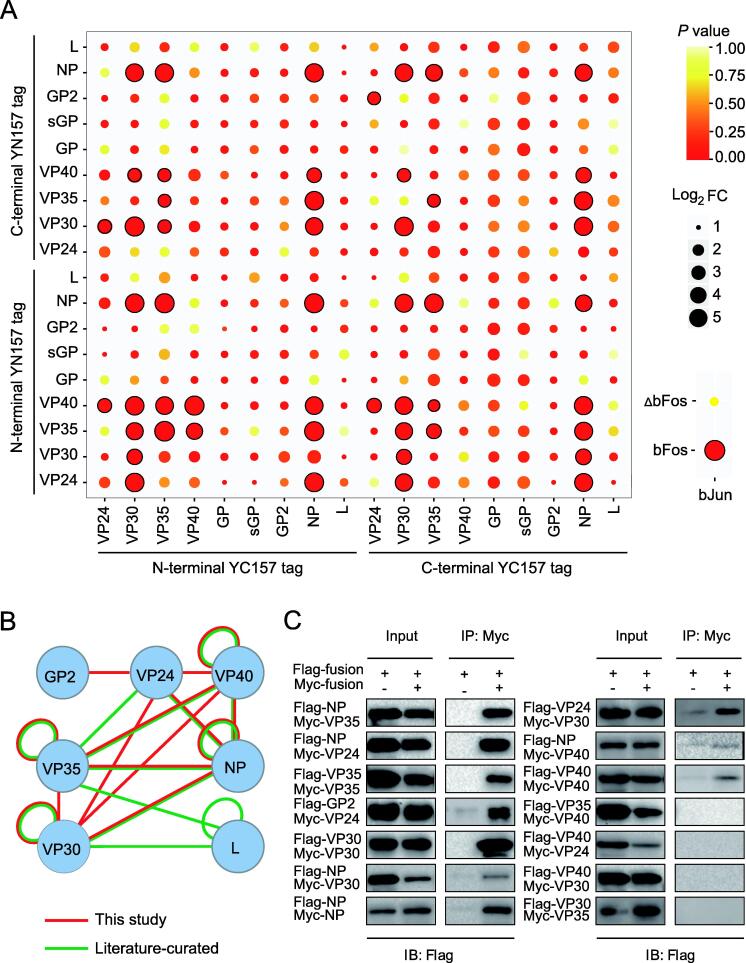
**Identification and validation ofEBOVintraviralPPIs separately by yEGFP-BiFC and Co-IP assay** **A.** An 18 × 18 cotransformation of yEGFP fragment-tagged EBOV proteins was carried out as indicated. Interactors are marked with black circles. bJun and bFos/ΔbFos were used as control indicators. Data are presented as mean ± SD of three biological replicates. *P* values were determined by Student’s *t*-test. **B.** Global intraviral PPI network of EBOV generated through the combination of our BiFC screening results with the literature-curated dataset. **C.** Validation of interactions among EBOV proteins by Co-IP assay. EBOV proteins fused with Flag or Myc tag were expressed in HEK293 cells. IP was performed using the anti-Myc antibody, and the co-immunoprecipitated proteins were detected using the anti-Flag antibaody. EBOV, Ebola virus; PPI, protein–protein interaction; IP, immunoprecipitation; IB, immunobotting; Co-IP, co-immunoprecipitation.

To exhaustively explore the PPI network of the nine encoded proteins of EBOV, we fused each protein with the YN157 or YC157 fragment in both orientations, yielding 36 fluorescent fragment-tagged proteins. After validation of their expression in yeast cells (Figure S2), pairs of these tagged proteins were cotransformed into yeast cells in an 18 × 18 matrix format in triplicate to screen intraviral PPIs of EBOV, and bJun and bFos/ΔbFos were used as controls. Transformed cells were analyzed by flow cytometry and fluorescence microscope (Figure S3). The ratio of fluorescent cells in each tested group was compared with that of the negative control group (bJun and ΔbFos), and those with a significant difference (*P* < 0.05) and a log_2_ fold change (test/control) greater than 2 were regarded as authentic PPIs. Finally, 51 interactions among 19 tagged proteins were obtained (Figure 2A). After removing redundancy, the dataset contained 14 interactions among 6 EBOV proteins, of which 9 interactions have been reported (Figure 2B). The interaction patterns of EBOV proteins with differently-oriented fluorescent fragment tags varied greatly by the BiFC method. The VP35–NP interaction was detected for all pairwise combinations of tagged VP35 and NP proteins, whereas the VP24–GP2 interaction was detected only for the VP24-YC157 and GP2-YN157 combination (Figure 2A, Figure S3D), indicating the advantage of using different fusion combinations to overcome steric hindrance to detect PPIs with the BiFC technique. Furthermore, by overexpressing Myc- and Flag-tagged EBOV proteins, we confirmed 10 of 14 interactions (71.4%) by co-immunoprecipitation (Co-IP) assay in mammalian cells (Figure 2C). Our new findings are consistent with the biological roles of the proteins; for example, the interactions of VP24–VP30 and VP24–GP2 suggest a role of VP24 in ribonucleoprotein (RNP) complex assembly and the release of GP2 by the virus. The interactions of VP40 with VP24 and VP30 also confirm that VP40 is involved in the release of viral particles from host cells. By integrating our EBOV-BiFC PPI network with the literature-curated dataset, a global intraviral EBOV PPI network containing 18 interactions among 7 proteins were obtained (Figure 2B) [17], [18], [19], [20], [21], [22], [23]. The comprehensive description of the interplay among EBOV proteins provides a basis for studying protein functions in terms of viral replication and particle secretion as well as for advancing novel therapeutic approaches.

### Novel p53 interactors revealed by BiFC-seq screening

To further show that the BiFC-seq method is suitable for high-throughput screening, the well-known tumor suppressor p53 was used as bait. Its interactors were screened against a universal human library fused with the N-terminal YN157 tag (YN157-cDNA library). We used HDM2, a known partner of p53, as a positive control [24]. A mutant form of HDM2 (ΔHDM2, with deletion of amino acids 1–139), which is incapable of binding with p53, was used as a negative control. Fluorescent cells were effectively distinguished between positive and negative controls by yEGFP-BiFC (Figure S4A and B). We also tested various linkers [25] between p53 or HDM2 and yEGFP halves by the yEGFP-BiFC method, and we found that the (GGGGS)3 linker produced the highest fluorescence ratio among the four tested linkers by flow cytometry (Figure S4C and D). To screen p53 interactors against the YN157-cDNA library, this library was sequentially transformed into competent yeast cells expressing p53 (YC157-p53 or p53-YC157) or corresponding controls with YC157 fragments (YC157-linker and linker-YC157). Each transformation was performed in triplicate. After 72 h of cultivation in SD-2 liquid medium, approximately 2000 fluorescent cells out of 1 × 10^8^ cells were obtained for each p53/control screening group by FACS sorting (Figure S5). This number is 10 to 100 times the number of positive colonies obtained with traditional Y2H screening.

**Figure 3:**
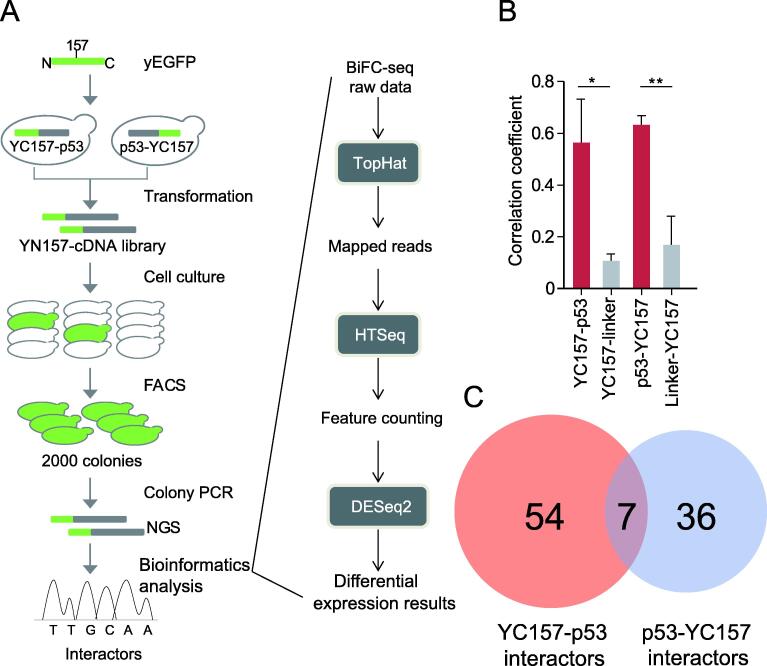
**Application of high-throughput screening using p53 as bait** **A.** Flowchart of the BiFC-seq screening. Fluorescence-positive cells were used as templates to amplify the genes encoding p53-binding partners. The PCR products were sequenced by NGS and analyzed with bioinformatics tools to identify the interactors of p53. **B.** Correlation analysis of the p53 and corresponding control screening groups. Data are presented as mean ± SD of three biological replicates. *, *P* < 0.05; **, *P* < 0.01 (Student’s *t*-test). **C.** Overlap of p53 interactors from the YC157-p53 and p53-YC157 screening groups. NGS, next-generation sequencing.

**Figure 4:**
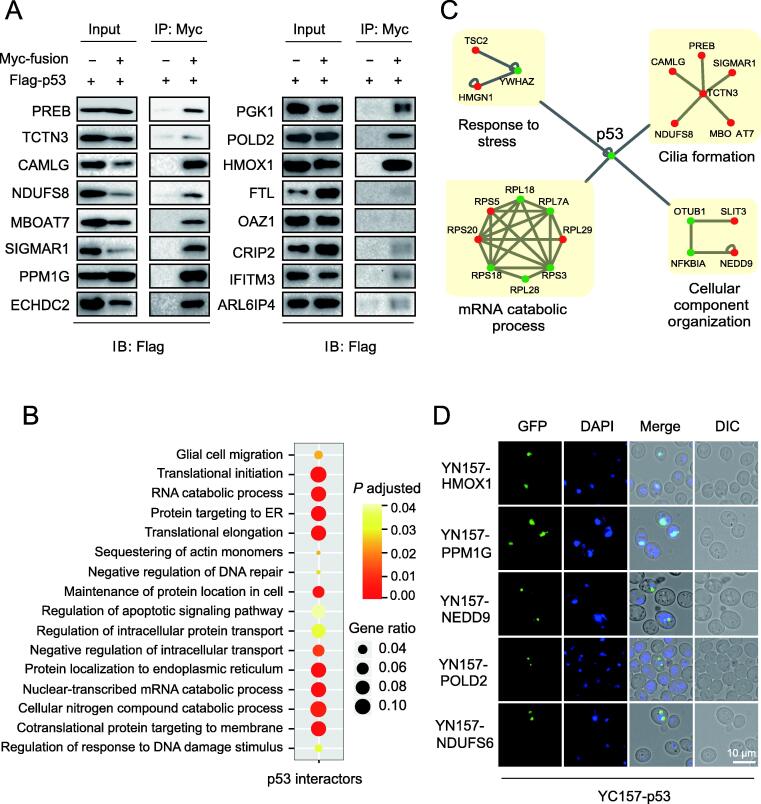
**Analysis and validation of p53 interactors obtained by BiFC-seq** **A.** Validation of the p53 interactors by Co-IP assays. p53 was expressed as a Flag-tagged fusion protein and its partners were expressed as Myc-tagged fusion proteins. HEK293 cells were transfected with the indicated constructs, immunoprecipitated with the anti-Myc antibody, and detected with the anti-Flag antibody. **B.** GO enrichment analysis showing the enriched biological processes for the p53 interactors obtained by BiFC-seq. **C.** Subnetworks constituted by p53 interactors from the BiFC-seq results. Green and red dots represent the p53 interactors that are reported and not reported in the BioGrid database, respectively. **D.** Validation of the interactions of p53 with its novel partners by yEGFP-BiFC. p53 was cotransformed with its interactors into yeast cells as indicated, and fluorescence was detected under a microscope 72 h after cotransformation. Scale bar, 10 μm.

To determine the identities of the positive colonies, PCR products of yeast colonies from each group were mixed and purified for NGS (Figure S6). After performing differential expression analysis with bioinformatics tools (Figure 3A), we identified 61 and 43 interactors for the YC157-p53 and p53-YC157 screening groups, respectively. The correlation coefficient of the RNA-seq results from p53 groups among triplicate screens was significantly higher than that of the corresponding controls (Figure 3B). There were seven overlapping interactions between the two groups (Figure 3C; Table S1). In total, 913 nonredundant interactors of p53 were recorded in the BioGrid database [26]. To our knowledge, this dataset is the most comprehensive dataset available, and we referred to these interactors as ‘known’ interactors. Twenty-one known p53 interactors were recaptured in this study. Thus, the sensitivity of BiFC-seq was ∼ 2% (21/913), which is similar to that of large-scale Y2H screening (∼ 1%) [27]. The specificity of BiFC-seq was evaluated by Co-IP assay. Among the 16 p53 interactors selected for validation, 11 were re-confirmed. Thus, the specificity of BiFC-seq was greater than 60% (11/16) (Figure 4A). The sampling sensitivity was estimated by three repeated screens of YC157-p53 and p53-YC157. In total, 61 interactors were identified for the YC157-p53 group. Among them, 45 interactors were consistently identified. The sampling sensitivity of YC157-p53 interactors was 73.8% per screen. A similar result was obtained for the p53-YC157 data, and the sampling sensitivity was 83.7%. Based on these data, we estimated that at least six screens are needed to reach 90% saturation (Figure S7). In addition, the screening results were evaluated with the PRINCESS tool, which was developed to assess the reliability of binary PPIs [28]. More than 64% of the interactions were of high confidence and had cut-off ratios greater than 2 (excluding the genes that were not recognizable) (Table S2). Then, we analyzed the functions of the p53 interactors by GO enrichment analysis. Consistent with previous reports, the p53 partners were found to participate in apoptosis, DNA damage response, translation, *etc.* (Figure 4B; Table S3). We further performed GO enrichment analysis of p53 by excluding 21 known p53 interactors. Most of the enriched GO terms were not changed, such as apoptosis, RNA catabolic process, and translation (Figure S8; Table S4). Four subnetworks of p53 interactors were enriched in the biological process category (Figure 4C), suggesting a mediating role of p53 among these subnetworks. The interactions of p53 with 5 interactors involved in the cilia formation subnetwork indicate that p53 might be associated with the division of centrosomes [29], which is consistent with previous findings in mouse cells [30], indicating the reliability of our screening results. Supporting the reproducibility of BiFC-seq, 45 of 61 interactors and 36 of 43 interactors were consistently identified in the three replicates of the YC157-p53 and p53-YC157 screening groups, respectively, with reproducibility of 73.8% (45/61) and 83.7% (36/43) individually. Some of the interactions of p53 with its novel partners were validated by BiFC assays (Figure 4D).

In three previous studies identifying p53 interactors by proteome-scale Y2H screening, only 51 interactions were detected and recorded in the BioGrid database (Table S5) [27], [31], [32]. Thus, the BiFC-seq approach had a higher sampling sensitivity than the traditional Y2H library or array screening approaches. Moreover, we found some p53-interacting membrane proteins that have a low possibility of being detected by the traditional Y2H method, such as calcium-modulating ligand (CAMLG), secretory carrier membrane protein 3 (SCAMP3), and interferon-induced transmembrane protein 3 (IFITM3).

The AP-MS method can be used to obtain protein complex information. Thus, we compared our data with that of a large-scale endogenous protein complex study [33]. A total of 50 high-confidence interactors of p53 were found by AP-MS. Among them, 13 were recorded in the BioGrid database (Table S6), while only 2 interactors were identified in our study. AP-MS identifies members of stable complexes whether they directly interact with the bait or not. In contrast to the AP-MS method, BiFC-seq identifies binary interactions between pairs of proteins and tends to enrich transient interactions. Stable binary interactions are also able to be identified by the BiFC-seq method, such as interactions between ribosomal proteins. The low overlap rate of the data from AP-MS and BiFC-seq might be due to the different features of the technologies.

### A genome-wide human interactome study by BiFC-seq

**Figure 5:**
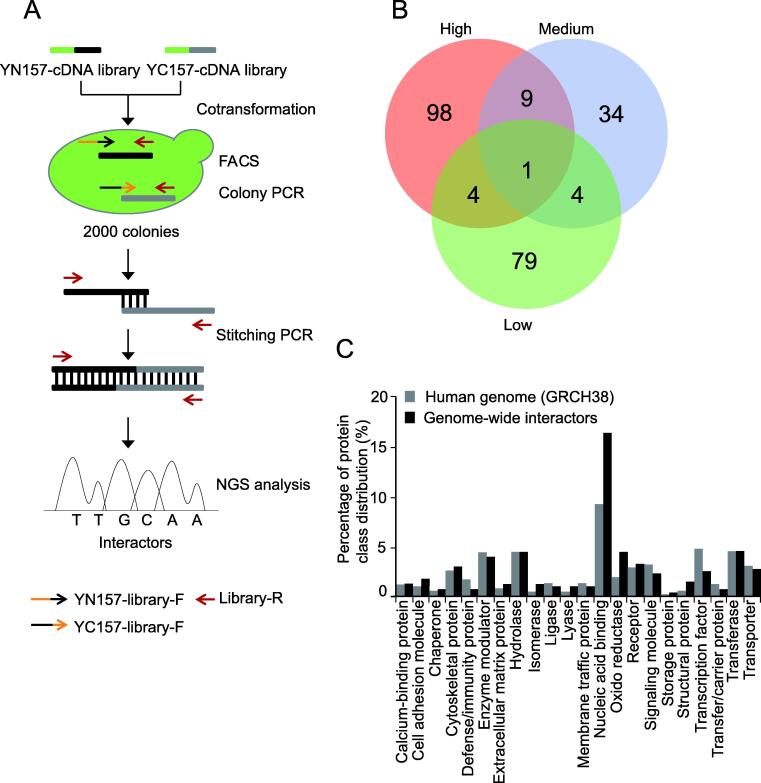
**Genome-widehumanPPIs revealed byBiFC-seq** **A.** Experimental workflow of genome-wide interactome screening. **B.** Venn diagram of overlapping interactions among three screening groups. **C.** Protein class distribution of genome-wide interactors along with that of the human genome.

**Figure 6:**
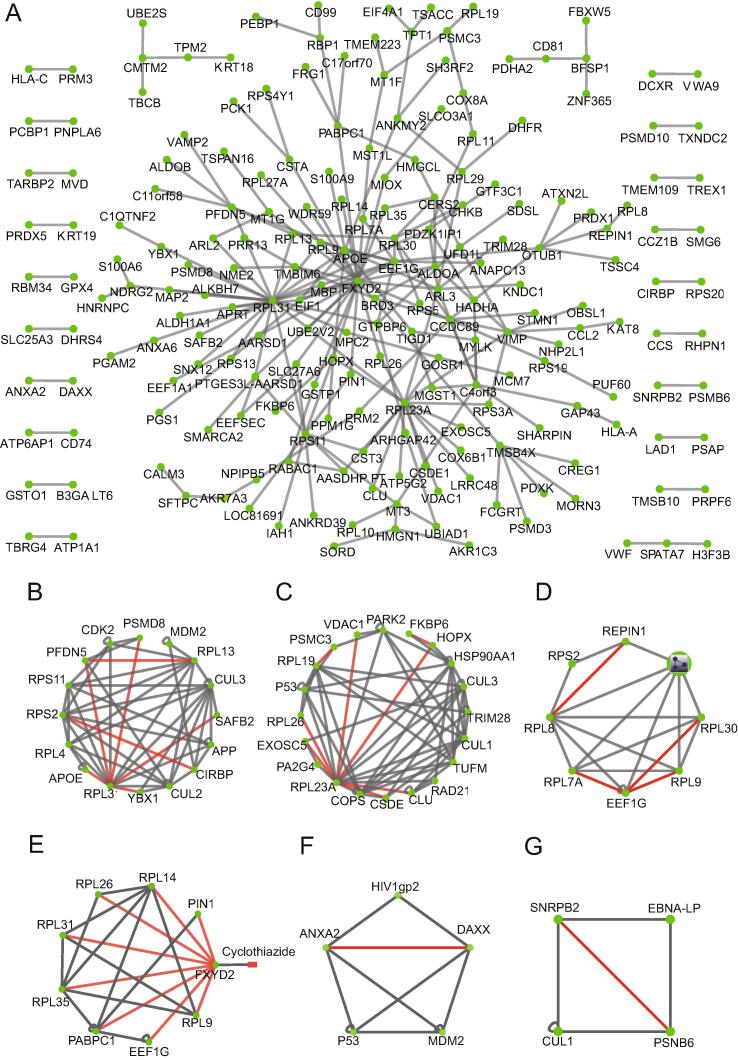
**Global PPI networkbased on BiFC-seq screening results** **A.** Global PPI network generated from the library-to-library screening results of BiFC-seq. **B.**–**G.** Various biological processes were presented by integrating BiFC-seq interactions with the BioGrid dataset, including gene expression (B), cellular metabolic process (C), biosynthetic process (D), protein metabolic process (E), viral infection process (F), and regulation of immune system process (G). Gray and red lines represent interactions from the BioGrid dataset and BiFC-seq results, respectively.

Based on the aforementioned results, we extended the BiFC-seq screening to a genome-wide scale. For this, we constructed a universal human library fused with the N-terminal YC157 tag (YC157-cDNA library). YN157-cDNA and YC157-cDNA libraries were cotransformed into AH109 competent yeast cells. After cultivation for 72 h in SD-2 liquid medium followed by FACS analysis, the fluorescent cells were divided into three groups (high, medium, and low) according to their fluorescence intensities, and approximately 2000 fluorescent cells were sorted from each group (Figure S9). Yeast colonies were picked as PCR templates until their diameters reached 2–3 mm. To identify the interacting partners within the same fluorescent yeast cell from library screening in a single NGS experiment, we joined the coding sequences (CDSs) of each interacting pair into one PCR amplicon using a stitching PCR strategy that consisted of two rounds of PCR (Figure 5A). In the first round, yeast colony PCR was conducted to amplify the CDSs of the interactors from the YN157-cDNA library and YC157-cDNA library individually. In the second round, amplicons from the first round were used as templates for stitching PCR to combine the amplicons via their corresponding complementary upstream primers (Figure S10). To exclude most non-specific PCR amplicons from the first round of PCR, stitching PCR products with more than 1000 bp fragments were mixed and purified for NGS analysis using the Illumina MiSeq platform. Approximately 1 million of ∼ 300 bp paired-end reads were generated from each group, and the stitching amplicons that contained the CDSs of the interactors from both libraries linked by complementary sequences were extracted and mapped to the CDS regions of the human reference genome (GRCH38). The protein pair with corresponding CDSs from the same read that were in-frame with the open reading frames (ORFs) were considered to be interactors. After removal of non-specific binding proteins which were detected by screening of the interactors against the YN157-cDNA library using the YC157 tag as a bait, a network was generated based on the PPIs from three screening groups, consisting of 229 interactions among 205 proteins (Figure 6A). Twelve of the interactions were reported in the BioGrid database (Table S7). A minority of the interactions overlapped among the three groups (Figure 5B), indicating that a relatively wide range of PPIs could be revealed via the classification of yeast cells with different fluorescence intensities. Among the 137 total interactions recognized by the PRINCESS tool, 26 had high confidence with a ratio cut-off of > 2 (Table S8). We classified these interactors with PANTHER [34]. Compared with all proteins encoded in the human genome, these interactors had similar functional distributions, which suggests that the BiFC-seq method has no bias toward certain PPI classes (Figure 5C). Furthermore, we found several clusters by superimposing the PPIs of this study onto the BioGrid dataset (Figure 6B–G), including gene expression, cellular metabolic process, biosynthetic process, protein metabolic process, viral infection process, and regulation of immune system process. As shown in Figure 6E, FXYD domain-containing ion transport regulator 2 (FXYD2), which was reported to interact only with the small molecule cyclothiazide in the BioGrid dataset [35], was found to participate in the protein metabolic process by interacting with translation-related partners. PPI resources are very useful for therapeutic discovery pursuits, such as drug repurposing. Potential drugs are identified by network-based methodologies. For example, dozens of candidate drugs have been prioritized for SARS-CoV-2 by using the interactome of virus between host [36], [37]. Interestingly, the ANXA2–DAXX and SNRPB2–PSMB6 connections were found to be involved in the viral infection process and the regulation of immune system process, respectively (Figure 6F and G). These results demonstrate the great potential of the BiFC-seq technique for genome-wide interactome screening.

## Conclusion

Here, we developed yEGFP-BiFC with high sensitivity and specificity in yeast cells. We used the yEGFP-BiFC to search the interactions among the nine encoded proteins of EBOV. By integrating our data with reported interactions, we generated a global EBOV intraviral PPI network, which provides new insights into EBOV pathogenesis from the perspective of the intraviral network. Next, we employed BiFC-seq for high-throughput PPI screening using p53 as a bait. We obtained a total of 97 p53 interactions, of which 21 were previously known. Furthermore, we extended the BiFC-seq method to a genome-wide interactome study and obtained 229 interactions of 205 proteins in a single screen. It should be noted that only limited interactions were identified for the human interactome by BiFC-seq. This was mainly due to the quality of cDNA used in the screening libraries and the number of positive clones that needed to be analyzed. To obtain more interactions using BiFC-seq, more colonies need to be identified in the future.

To evaluate the co-expression performance of interactors identified by BiFC-seq, we compared our results with non-interacting protein pairs from the Negatome dataset [38] and with the transcriptomic data of 74 tissues from the Human Protein Atlas (HPA) database [39]. The percentages of co-expressed interactors of p53 and genome-wide interactors were 20.8% and 13.1%, respectively, while for the Negatome data, the value was 11.4% (Table S9). These results demonstrate that the BiFC-seq interactors are more likely to be co-expressed than non-interacting proteins.

Compared with those identified by genetic approaches, such as gene-trap insertional mutagenesis [40], RNAi [41], and CRISPR/Cas9 screening [42], the PPIs identified by BiFC-seq are mostly physical and binary. The interactions from these two kinds of methods are complementary. The recently developed CrY2H-seq method is a highly scalable screening approach to detect binary PPIs in yeast [43]; similar to the Y2H method, it employs more than one reporter with stringent selection for PPIs. However, self-activated baits cannot be screened by CrY2H-seq. In contrast, no self-activated baits need to be excluded in BiFC-seq owing to the use of a single EGFP reporter, which might also increase the number of false positives.

In summary, we have developed a BiFC-seq method that is amenable to high-throughput PPI screening. This technique is highly sensitive for the screening of physical binary PPIs and can be easily applied to other large-scale interactome mapping studies.

## Data availability

The NGS data for p53 and genome-wide screening have been deposited in the Genome Sequence Archive [44] at the National Genomics Data Center, Beijing Institute of Gemonics, Chinese Academy of Sciences / China National Center for Bioinformation (GSA: HRA000766), and are publicly accessible at https://ngdc.cncb.ac.cn/gsa.

## CRediT author statement

**Limin Shang:** Methodology, Writing - original draft, Visualization, Writing - review & editing, Funding acquisition. **Yuehui Zhang:** Validation. **Yuchen Liu:** Data curation. **Chaozhi Jin:** Investigation. **Yanzhi Yuan:** Investigation. **Chunyan Tian:** Investigation. **Ming Ni:** Resources. **Xiaochen Bo:** Resources. **Li Zhang:** Investigation. **Dong Li:** Data curation. **Fuchu He:** Supervision, Project administration. **Jian Wang:** Supervision, Project administration, Funding acquisition, Writing - review & editing, Visualization. All authors have read and approved the final manuscript.

## Competing interests

The authors declare no competing financial interests.

## Supplementary material

Supplementary File S1 **Yeast based BiFC-seq protocol**

Supplementary Figure S1 **Expression of yEGFP in yeast cells** Intact yEGFP or YN157/YC157 fragments were transformed into yeast cells that were then cultured at 30  °C for 24 h in SD-2 medium and incubated at 4 °C for approximately 48 h for fluorophore maturation before observation, scale bar: 10 μm. WL, whole length.

Supplementary Figure S2 **Expression of EBOV proteins fused with fluorescent fragments** N-terminal flag tagged EBOV proteins fused with fluorescent fragments as indicated, expression of fused protein is detected by anti-flag antibody.

Supplementary Figure S3 **Validation of intra EBOV PPIs by yEGFP-BiFC A.–N.** To validate the intra EBOV PPIs detected by flowcytometry, EBOV proteins with yEGFP fragment tags were cotransformed into yeast cells, the fluorescence of cells was observed under the fluorescent microscope.

Supplementary Figure S4 **Validation of the interaction between HDM2 and p53 using yEGFP-BiFC A.** p53 and HDM2/ΔHDM2 were cotransformed into yeast cells as indicated, the fluorescent yeast cells were observed under fluorescence microscope, scale bar: 10 μm **B.** fluorescent cells from A were analyzed by flow cytometry. **C.** List of different peptides that used to link YN157 or YC157 with HDM2 or p53. **D.** Different linkers between YN157-HDM2 and YC157-p53 as indicated were check for their fluorescent cell ratio 72 h after cotransformation by flow cytometry. Data are presented as means ± SD of three biological replicates. Error bars: s.d. *, *P* value <0.05, n.s. non-significant.

Supplementary Figure S5 **Yeast cells containing interactors of p53 screened by yEGFP-BiFC method were sorted by FACS A.–D.** The YN157-tagged human universal library was sequentially transformed into yeast cells containing p53 and control groups, including YC157-p53 (A), p53-YC157 (B), YC157-linker (C), and linker-YC157 (D). The fluorescent cells were sorted 72 h after transformation. Approximately 2000 fluorescent cells were sorted from the P2 gate of each group.

Supplementary Figure S6 **Yeast colony PCR A.** Fluorescent yeast colonies were used as templates to amplify the cDNA inserts. A portion of the PCR products was used for electrophoresis as indicated. **B.** The electrophoresis results of the purified PCR products for each group.

Supplementary Figure S7 **Number of interactions detected after each screen using p53 as bait by BiFC-seq A.** Number of YC157-p53 interactors from three rounds of screening. **B.** Number of p53-YC157 interactors from three rounds of screening.

Supplementary Figure S8 **Enrichment of biological processes for p53 unreported interactors from the screening results**

Supplementary Figure S9 **Yeast cells containing genome-wide interactors screened by the yEGFP-BiFC method were sorted by FACS A.−C.** Yeast cells that contain binding partners from YN157-ΔHDM2 + YC157-p53 (A), YN157-HDM2 + YC157-p53 (B), and YN157-library + YC157-library (C) were sorted out into three groups according to their fluorescence intensities.

Supplementary Figure S10 **Electrophoresis of Stitching PCR products** Gel electrophoresis of stitching PCR products. N-library and C-library represent yeast colony PCR product derivated from YN157-library and YC157-library of the same fluorescent cell, N-library + C-library represents stitching PCR product of N-library and C-library. Mr: 15,000 marker.
