# Supplementary material for: A Yeast BiFC-seq Method for Genome-wide Interactome Mapping
Source: Genomics Proteomics Bioinformatics. 2021 Jul 24;20(4):795–807. doi: 10.1016/j.gpb.2021.02.008 (PMC9880813; doi:10.1016/j.gpb.2021.02.008)
Supplement: Supplementary File S1 — Yeast based BiFC-seq protocol [file mmc1.docx]

**BiFC-seq protocol**

**Reagents**

| **Reagent** | **Manufacture** | **Catalog ID** |
| --- | --- | --- |
| Yeast Extract | Thermo Fisher Scientific | LP0021 |
| Peptone | Thermo Fisher Scientific | LP0049B |
| Adenine hemisulfate | Sigma | A3159 |
| Agar | Thermo Fisher Scientific | LP0011B |
| Yeast synthetic drop-out medium supplements | Sigma | Y1376, Y0750 |
| Lithium acetate dihydrate | Sigma | L6883 |
| PEG 3350 | Sigma | P3640 |
| Salmon sperm DNA | Sigma | D1626 |

**Culture medium and buffers**

| **Culture medium and buffers** | **Components** |
| --- | --- |
| **LB** | Tryptone: 10 g, Yeast Extract: 5 g, NaCl: 10 g, dissolved in 1 L distilled water |
| **YPAD** | Yeast Extract: 10 g, Peptone: 20 g, Glucose: 20 g, Ade: 0.4 g, dissolved in 1 L distilled water |
| **SD-L** | YNB: 6.7 g, Glucose: 20 g, DO Supplement-Leu: 0.69 g, pH 5.8, dissolved in 1L distilled water |
| **SD-2** | YNB: 6.7 g, Glucose: 20 g, DO Supplement-Leu/-Trp: 0.64 g, pH 5.8, dissolved in 1L distilled water |
| **10×TE** | 100 mM Tris-HCl (pH 7.5), 10 mM EDTA |
| **TE/LiAc (10 ml)** | 1 ml 10×TE, 1 ml 10**×**LiAC(1 M), 8 ml distilled water |
| **PEG/LiAc (10 ml)** | 8 ml 50% PEG3350, 1 ml 10**×**TE, 1 ml 1M LiAc |

| **Plasmids and Primers** | | |
| --- | --- | --- |
| **Plasmid** | **Primer** | **Sequence** |
| pPC86-pADH-linker | pPC86-pADH-F | 5’-AAAGGTACCATCCGGGATCGAAGAAATG-3’ |
|  | pPC86-linker-R | 5’-CTAGACTAGTAGAACCACCACCACCAGAACCACCACCACCAGAACCACCACCACCAGATCTGAATTCCCGGGGTCGACGGCATAAGCTTGGAGTTGATT-3’ |
| pDBLeu-pADH-linker | pDBLeu-CYH2-pADH-F | 5’- GGAAGATCTTACCGTAACCGGCTG-3’ |
|  | pDBLeu-linker-R | 5’-CTAGACTAGTCTAGAAGAACCACCACCACCAGAACCACCACCACCAGAACCACCACCACCCCATGGGCTAGCACCCGGGGTCGACATTGCTTCAAGCTTGGAGTTGATT-3’ |
| pPC86-YN157-bJun | YN157-F | 5’-GCCGTCGACCATGTCTAAAGGTGAAGAATTAT-3’ |
|  | YN157-R | 5’-GCCAGATCTTTGTTTGTCAGCCATGATGTAAA-3’ |
|  | bJun-F | 5’-ATTACTAGTAAGGCGGAGAGGAAGCGCATGAG-3’ |
|  | bJun-R | 5’-ATTGCGGCCGCGTGGTTCATGACTTTCTGTTT-3’ |
| pPC86-bJun-YN157 | bJun-F | 5’-ATTGTCGACTAAGGCGGAGAGGAAGCGCATGAG-3’ |
|  | bJun-R | 5’-GCCAGATCTGTGGTTCATGACTTTCTGTTT-3’ |
|  | YN157-F | 5’-GCCACTAGTATGTCTAAAGGTGAAGAATTA-3 |
|  | YN157-R | 5’-ATTGCGGCCGCTTGTTTGTCAGCCATGATGTA-3’ |
| pDBLeu-YC157-bFos/△bFos | YC157-F | 5’-GCCGTCGACGATGAAGAATGGTATCAAAGTTAACT-3’ |
|  | YC157-R | 5’-CGGCCATGGTTTGTACAATTCATCCATACCAT-3’ |
|  | bFos-F | 5’-TAATCTAGAGGTCGTGCGCAGTCCATCGGTC-3 |
|  | bFos-R | 5’-ATTGCGGCCGCACCCAGGTCGTTCGGGATTTTGC-3’ |
|  | △bFos-F | 5’-TAATCTAGAGGTCGTGCGCAGTCCATCGGTC-3’ |
|  | △bFos-R | 5’-ATTGCGGCCGCACCCAGGTCGTTCGGGATTTTGC-3’ |
| pDBLeu-bFos-YC157 | bFos-F | 5’-TAAGTCGACTGGTCGTGCGCAGTCCATCGGT-3’ |
|  | bFos-R | 5’-ATTCCATGGACCCAGGTCGTTCGGGATTTT-3’ |
|  | YC157-F | 5’-GCCTCTAGAATGAAGAATGGTATCAAAGTTAAC-3’ |
|  | YC157-R | 5’-ATTGCGGCCGCTTTGTACAATTCATCCATACC-3’ |
| pDBLeu-YC157-p53 | p53-F | 5’-ACCTCTAGAATGGATGATTTGATGCTGTCCC-3’ |
|  | p53-R | 5’-ATTTGCGGCCGCGTCTGAGTCAGGCCCTTCTGT-3’ |
| pDBLeu-p53-YC157 | p53-F | 5’-ATTGTCGACCATGGATGATTTGATGCTGTCCC-3’ |
|  | p53-R | 5’-ATTGCTAGCGTCTGAGTCAGGCCCTTCTGT-3’ |
| pPC86-YN157-HDM2/△HDM2 | HDM2-F1 | 5’-GCCACTAGTATGTGCAATACCAACATGTCTG-3’ |
|  | HDM2-R1 | 5’-ATTGCGGCCGCGGGGAAATAAGTTAGCACAATCA-3’ |
|  | △HDM2-F1 | 5’-TGCACTAGTATCTACAGGAACTTGGTAG-3’ |
|  | △HDM2-R | 5’-ATTGCGGCCGCGGGGAAATAAGTTAGCACAATCA-3’ |

**Construction of yEGFP-BiFC vectors**

pPC86-ADH-linker

To remove the coding sequences of NLS-GAL4 AD, pPC86 vector was digested with *Kpn* I and *Spe* I, pPC86-pADH-F and pPC86-linker-R primers were used to amplify pADH-(GGGGS)3 sequence using pPC86 as template and ligated with digested pPC86 vector, yielding pPC86-pADH-linker


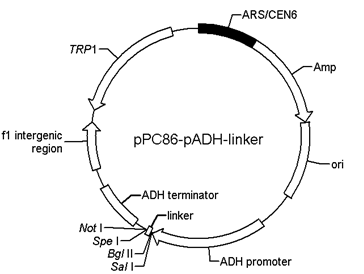


**pDBLeu-ADH-linker**

To remove the coding sequences of GAL4 BD, pDBLeu vector was digested with *Bgl* II and *Spe* I, pDBLeu-CYH2-pADH-F and pDBLeu-linker-R primers were used to amplify CYH2- pADH-(GGGGS)3 sequence using pDBLeu as template and ligated with digested pDBLeu vector, yielding pDBLeu-pADH-linker


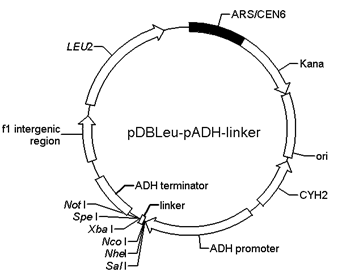


pPC86-YN157-bJun, pPC86-bJun-YN157, pDBLeu-YC157-bFos/ΔbFos, pDBLeu-bFos-YC157, pDBLeu-YC157-p53, pDBLeu-p53-YC157 and pPC86-YN157-HDM2/ΔHDM2 plasmids were constructed using corresponding primers and ligated into pPC86-pADH-linker and pDBLeu-pADH-linker vectors

**Protocol**

**Yeast Transformation Protocols** [[1](#_ENREF_1)]

1. 5 ml YPAD or SD-Leu medium was inoculated with several 2–3 mm yeast colonies.

2. Transfer yeast cells to a ﬂask containing 50 ml YPAD or SD-Leu medium, incubate at 30°C for about 16 hr with continuous shaking (220 rpm).

3. The overnight cultures were transferred into 300 ml YPAD medium and incubated at 30°C for about 3 hr with shaking (220 rpm) until the OD_600_ reaches 0.5 ± 0.1.

4. Cultured cells were collected in 500 ml tubes and pelleted at 1000 g for 10 min at room temperature.

5. The supernatant was discarded, and cell pellets were resuspended with 50 ml sterile TE.

6. Centrifuge the cells at 1000 g for 10 min and discard the supernatant.

7. Add the following volume of mixtures to the yeast component cells; for the large scale screening, add 100 µg plasmid; for the library scale screening, add 200 µg plasmid.

| Transformation mix components | Volume (µl) |
| --- | --- |
| PEG 3350 (50% (w/v)) | 2400 |
| LiAc 1.0 M | 360 |
| Single-stranded carrier DNA (2.0 mg/ml) | 500 |
| Plasmid DNA plus sterile water | 340 |
| Total volume | 3600 |

8. Incubate the mixtures at 30°C for 30 min with shaking (220 rpm), then heat shock for 15 min at 42°C in the water bath, swirl every 5 min to mix, chill the cells on ice for 2 min.

9. Centrifuge cells for 5 min at room temperature, discard the supernatant, and resuspend cells in 10 ml 1**×**TE for plating.

**Screening p53 interactors using BiFC-seq**

1. AH109 yeast component cells were transformed with 10 μg pDBLeu-YC157-p53, pDBLeu-p53-YC157, pDBLeu-YC157-linker, and pDBLeu-linker-YC157 plasmids, and plate transformed cells on SD-Leu plates.
2. Yeast component cells containing p53 or vector plasmids were transformed with 100 µg pPC86-YN157-library plasmid.
3. After transformation, cells were resuspended in 10 ml of liquid SD without tryptophan and leucine (SD-2) medium, cultured in a 30°C shaker for 24 hours, and incubated at 4°C for an additional 48 hours for fluorophore maturation.
4. The yeast cells were collected by centrifugation at 7000 rpm for 30 s and resuspended with PBS; cells expressing reconstituted yEGFP were sorted by FACS.
5. The sorted yeast cells were collected with PBS, spread onto SD-2 plates, and cultured in a 30°C incubator for 2 days until yeast colonies reached 2−3 mm in diameter.
6. Pick up yeast colonies and digested with 20 μl 0.02 N NaOH. After 5 minutes of boiling, 2 μl of the solution was used as a template to amplify the inserted cDNA.
7. For each amplification, 3 μl of the PCR products was mixed and purified using the QIAquick PCR Purification Kit.
8. The purified PCR products were sheared with a Covaris S220 system and sequenced by NGS.
9. The filtered reads were mapped to the GRCh38 genome by TopHat. The raw counts of the sequencing reads for each transcript were calculated by the HTSeq Python package. DESeq2 was then used for differential expression analysis between p53 and control groups. These with significant differences (p.adj < 0.05) were regarded as p53 interactors.

**Screening genome-wide interactors using BiFC-seq**

1. AH109 yeast component cells were cotransformed with 200 μg of pPC86-YN157-library and pDBLeu-YC157-library.
2. After transformation, cells were resuspended in 20 ml of liquid SD without tryptophan and leucine (SD-2) medium, cultured in a 30°C shaker for 24 hours, and incubated at 4°C for an additional 48 hours for fluorophore maturation.
3. The yeast cells were collected by centrifugation at 7000 rpm for 30 s and resuspended with PBS; cells expressing reconstituted yEGFP were sorted by FACS.
4. The sorted yeast cells were collected with PBS, spread onto SD-2 plates, and cultured in a 30°C incubator for 2 days until yeast colonies reached 2−3 mm in diameter.
5. Yeast colonies were used as templates to amplify encoding sequences of interactors from YN157-library and YC157-library individually; amplicons were used as templates to stitch PCR products together.
6. For each amplicon, 3 μl of the PCR products were mixed with more than 1000 bp fragments were purified for the NGS analysis.
7. The stitching amplicons that contain encoding sequences of interactors from both library pools linked by complementary sequences were extracted and mapped to the coding sequence (CDS) regions of the human reference genome.
8. Both of the gene encoding sequences from the same read that are in frame with the open reading frame (ORF) were considered to be interactors.

**References**

[1] Gietz RD, Schiestl RH. High-efficiency yeast transformation using the LiAc/SS carrier DNA/PEG method. Nat Protoc 2007;2:31−4.
