# Supplementary figures and images for: A Yeast BiFC-seq Method for Genome-wide Interactome Mapping
Source: Genomics Proteomics Bioinformatics. 2021 Jul 24;20(4):795–807. doi: 10.1016/j.gpb.2021.02.008 (PMC9880813; doi:10.1016/j.gpb.2021.02.008)

yEGFP:WL

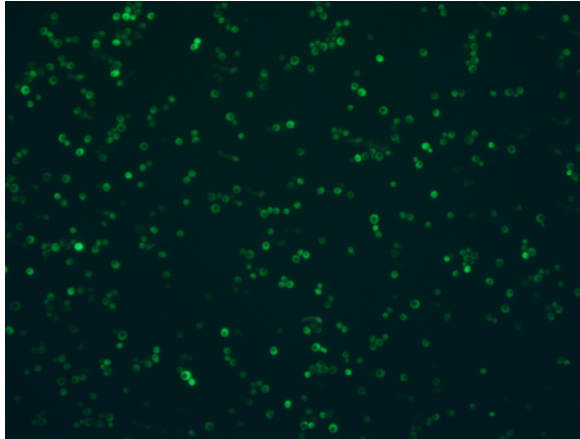

yEGFP:YN157+YC157

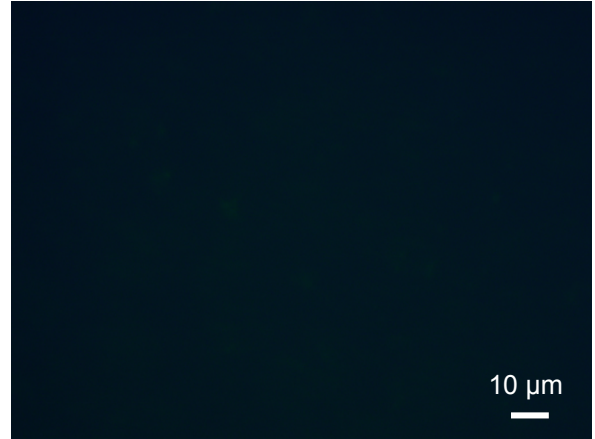

Supplement: Supplementary Figure S1 — Expression of yEGFP in yeast cells Intact yEGFP or YN157/YC157 fragments were transformed into yeast cells that were then cultured at 30 °C for 24 h in SD-2 medium and incubated at 4 °C for approximately 48 h for fluorophore maturation before observation, scale bar: 10 μm. WL, whole length. [file mmc2.pdf]

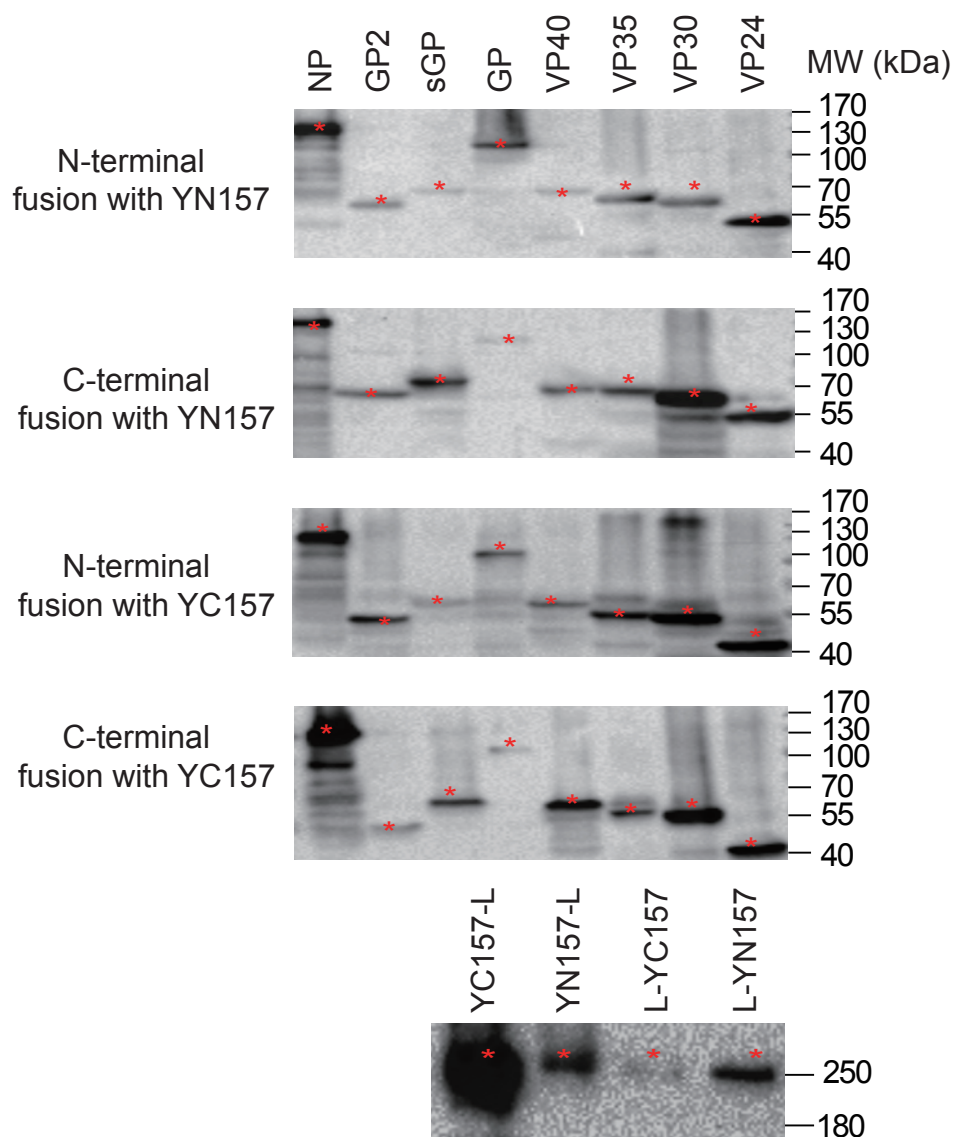

Supplement: Supplementary Figure S2 — Expression of EBOV proteins fused with fluorescent fragments N-terminal flag tagged EBOV proteins fused with fluorescent fragments as indicated, expression of fused protein is detected by anti-flag antibody. [file mmc3.pdf]

A

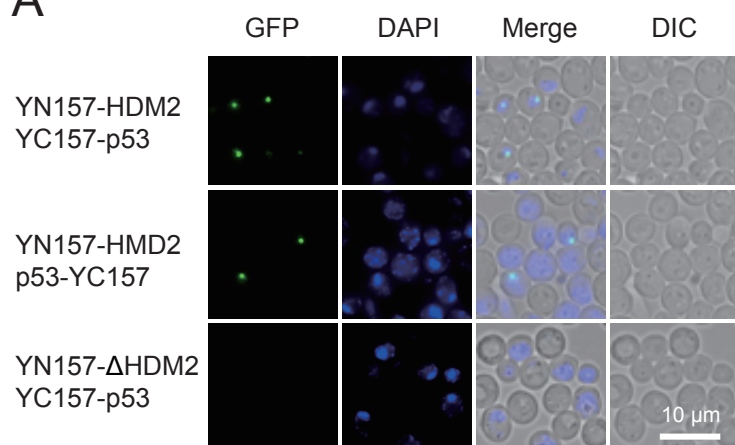

C

| Linker  | Sequence        |
|---------|-----------------|
| Linker1 | (GGGGS)3        |
| Linker2 | (GGGGS)2        |
| Linker3 | RSIAT           |
| Linker4 | RPACKIPNDLQKVMN |

B

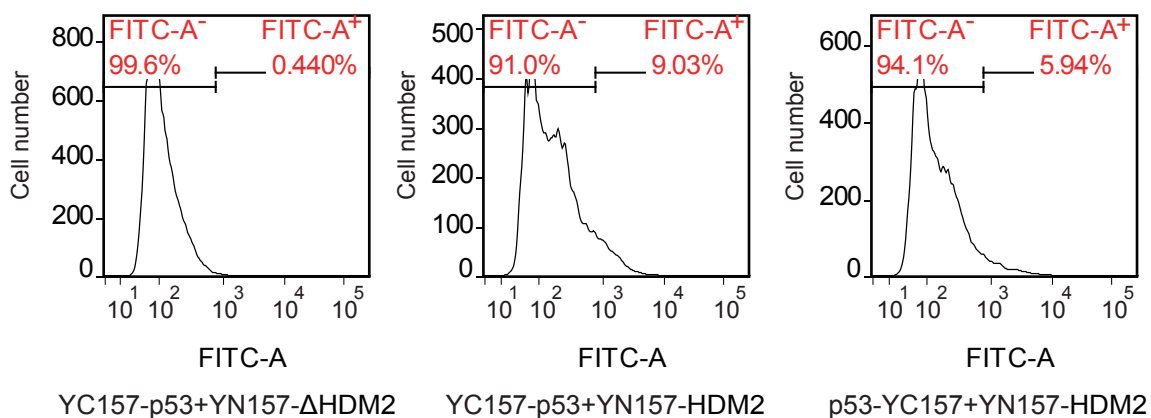

D

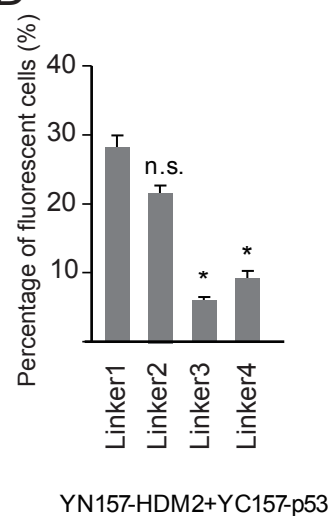

Supplement: Supplementary Figure S4 — Validation of the interaction between HDM2 and p53 using yEGFP-BiFC A. p53 and HDM2/ΔHDM2 were cotransformed into yeast cells as indicated, the fluorescent yeast cells were observed under fluorescence microscope, scale bar: 10 μm B. fluorescent cells from A were analyzed by flow cytometry. C. List of different peptides that used to link YN157 or YC157 with HDM2 or p53. D. Different linkers between YN157-HDM2 and YC157-p53 as indicated were check for their fluorescent cell ratio 72 h after cotransformation by flow cytometry. Data are presented as means ± SD of three biological replicates. Error bars: s.d. *, P value <0.05, n.s. non-significant. [file mmc5.pdf]

A

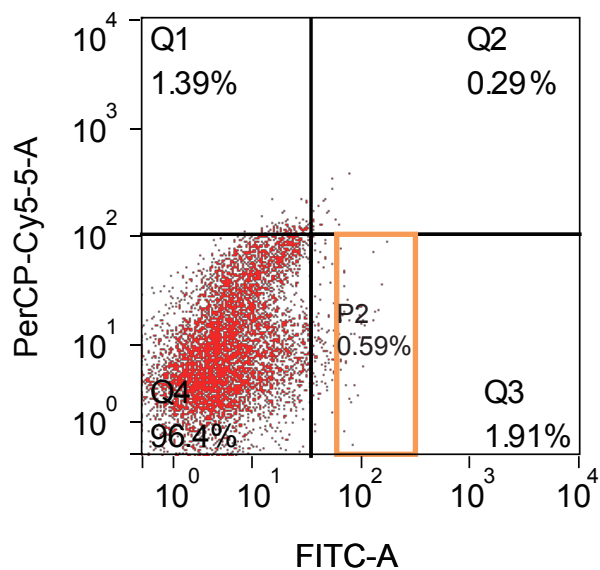

B

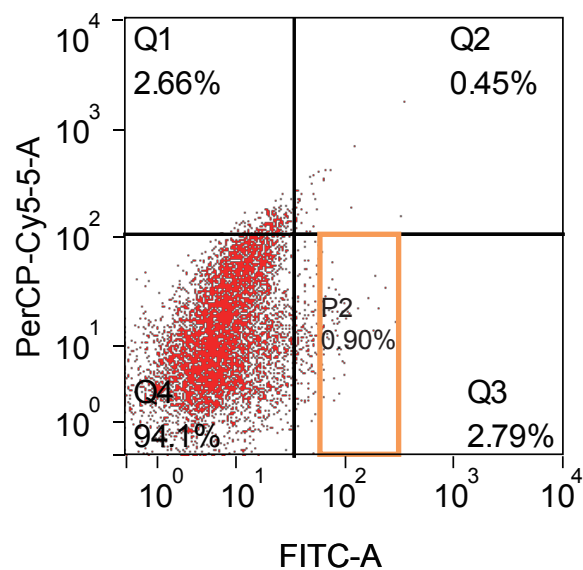

C

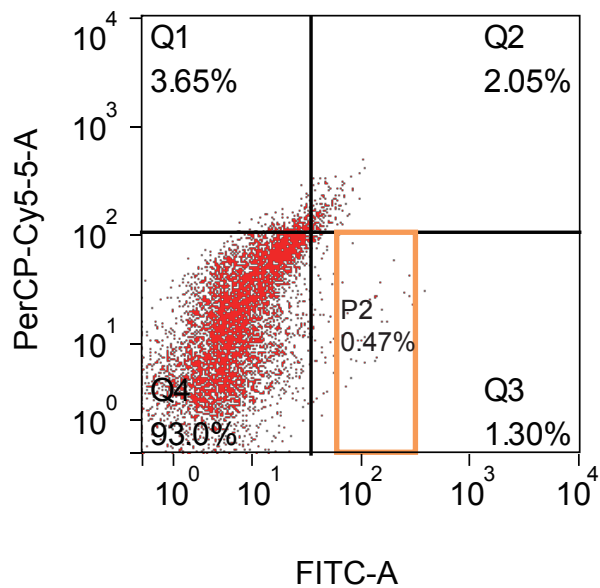

D

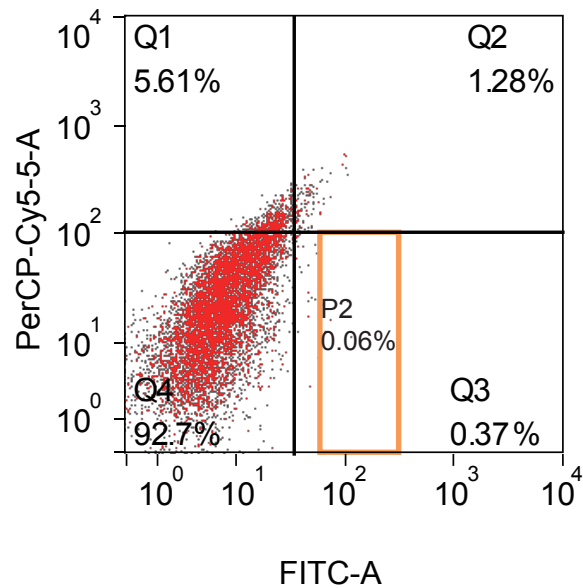

Supplement: Supplementary Figure S5 — Yeast cells containing interactors of p53 screened by yEGFP-BiFC method were sorted by FACS A.–D. The YN157-tagged human universal library was sequentially transformed into yeast cells containing p53 and control groups, including YC157-p53 (A), p53-YC157 (B), YC157-linker (C), and linker-YC157 (D). The fluorescent cells were sorted 72 h after transformation. Approximately 2000 fluorescent cells were sorted from the P2 gate of each group. [file mmc6.pdf]

A

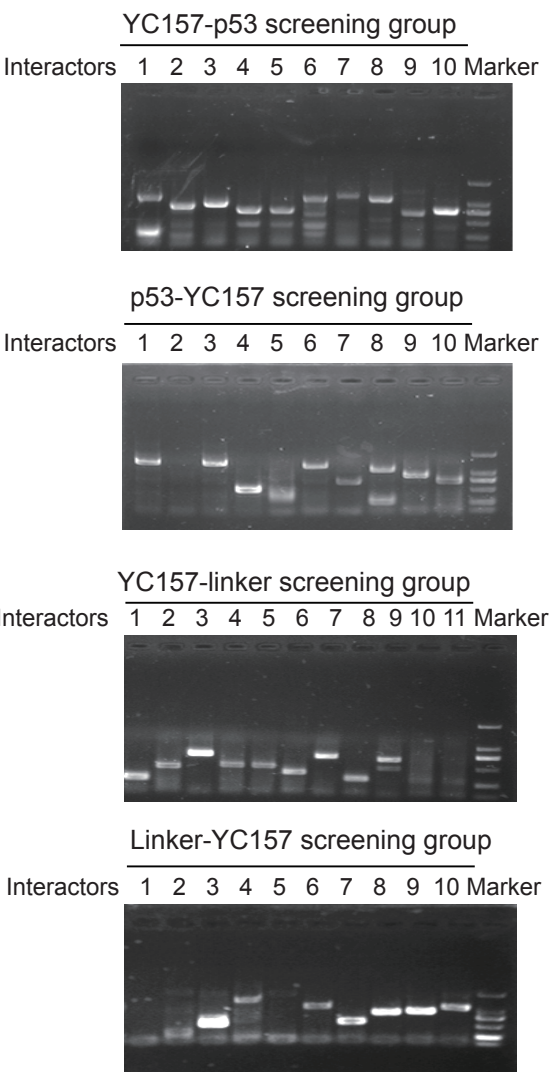

B

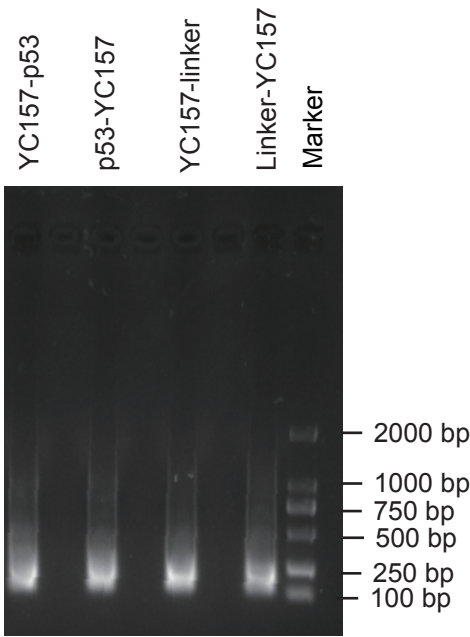

Supplement: Supplementary Figure S6 — Yeast colony PCR A. Fluorescent yeast colonies were used as templates to amplify the cDNA inserts. A portion of the PCR products was used for electrophoresis as indicated. B. The electrophoresis results of the purified PCR products for each group. [file mmc7.pdf]

A

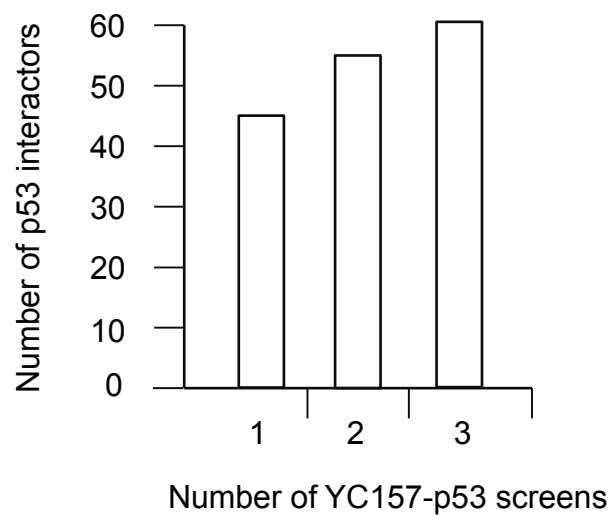

B

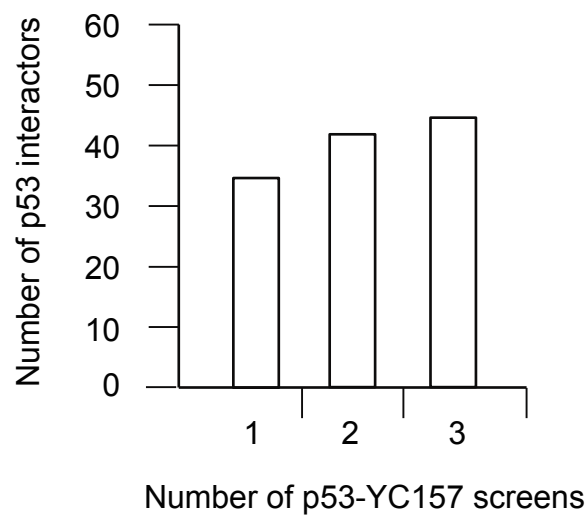

Supplement: Supplementary Figure S7 — Number of interactions detected after each screen using p53 as bait by BiFC-seq A. Number of YC157-p53 interactors from three rounds of screening. B. Number of p53-YC157 interactors from three rounds of screening. [file mmc8.pdf]

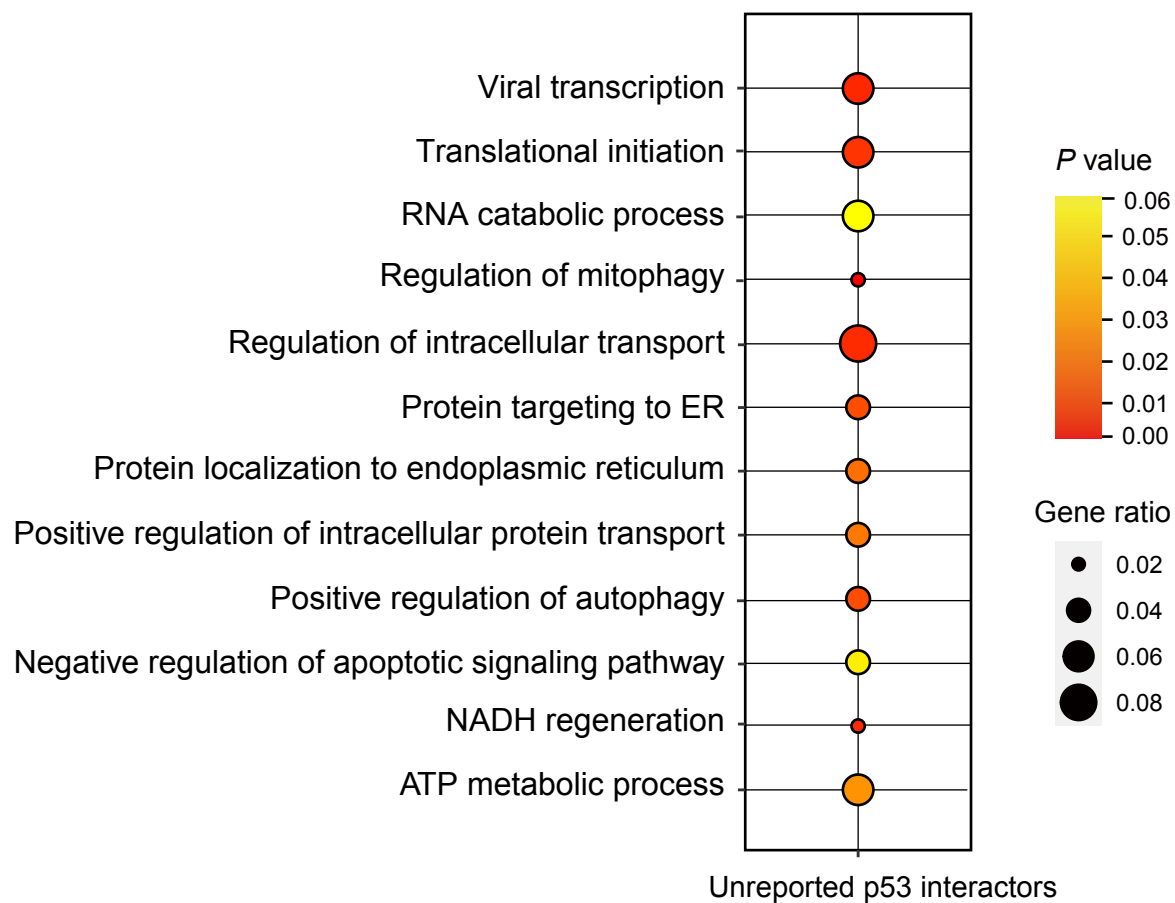

Supplement: Supplementary Figure S8 — Enrichment of biological processes for p53 unreported interactors from the screening results [file mmc9.pdf]

A

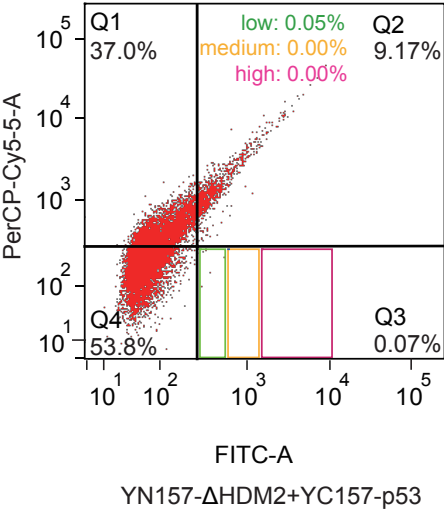

B

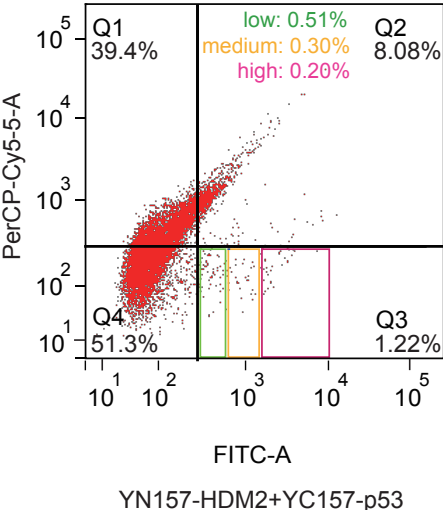

C

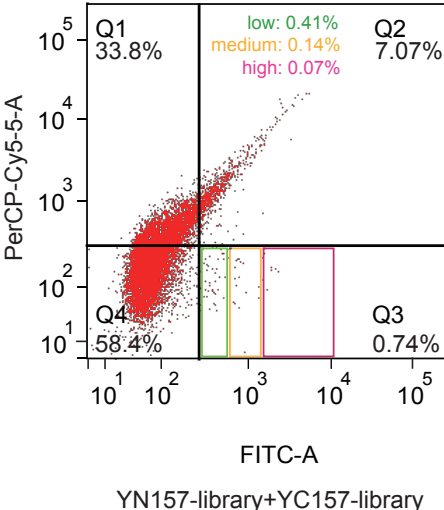

Supplement: Supplementary Figure S9 — Yeast cells containing genome-wide interactors screened by the yEGFP-BiFC method were sorted by FACS A.−C. Yeast cells that contain binding partners from YN157-ΔHDM2 + YC157-p53 (A), YN157-HDM2 + YC157-p53 (B), and YN157-library + YC157-library (C) were sorted out into three groups according to their fluorescence intensities. [file mmc10.pdf]

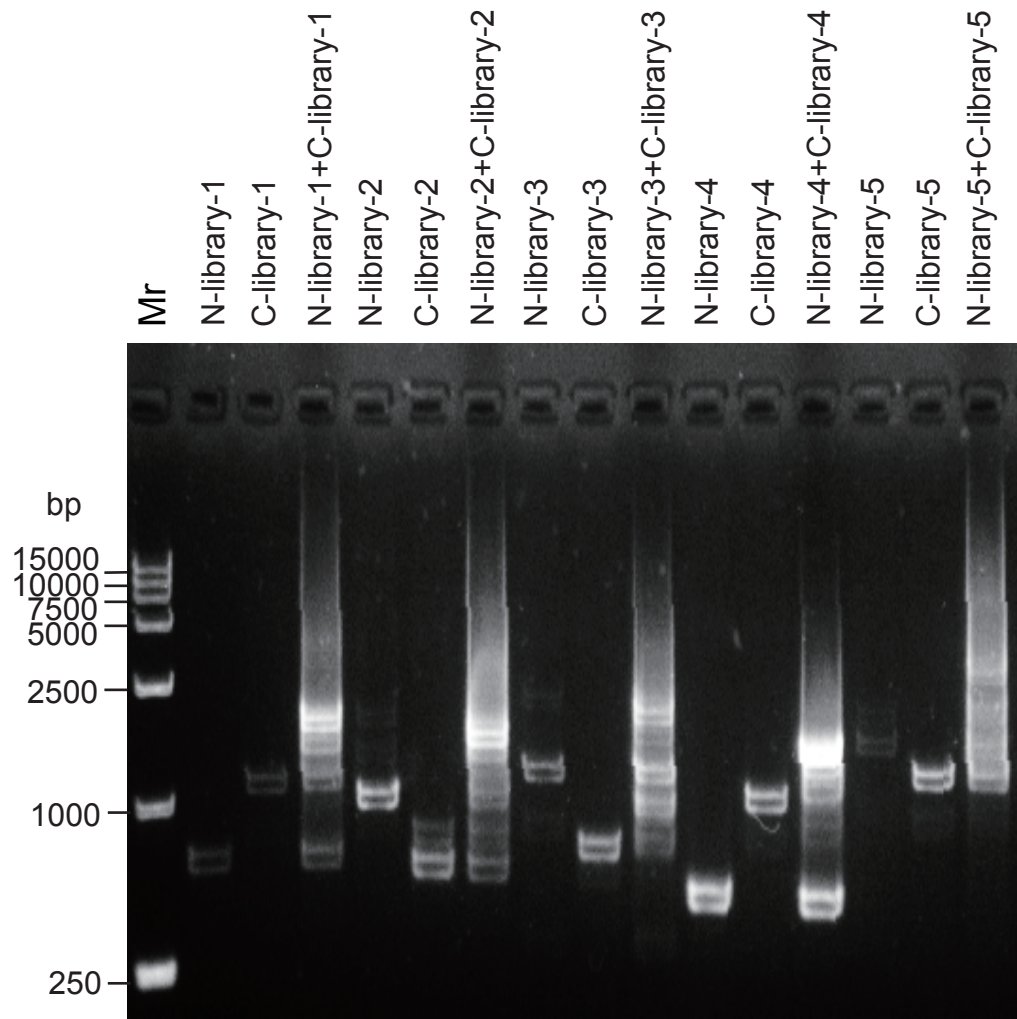

Supplement: Supplementary Figure S10 — Electrophoresis of Stitching PCR products Gel electrophoresis of stitching PCR products. N-library and C-library represent yeast colony PCR product derivated from YN157-library and YC157-library of the same fluorescent cell, N-library + C-library represents stitching PCR product of N-library and C-library. Mr: 15,000 marker. [file mmc11.pdf]
