# Supplementary material for: A Yeast BiFC-seq Method for Genome-wide Interactome Mapping
Source: Genomics Proteomics Bioinformatics. 2021 Jul 24;20(4):795–807. doi: 10.1016/j.gpb.2021.02.008 (PMC9880813; doi:10.1016/j.gpb.2021.02.008)

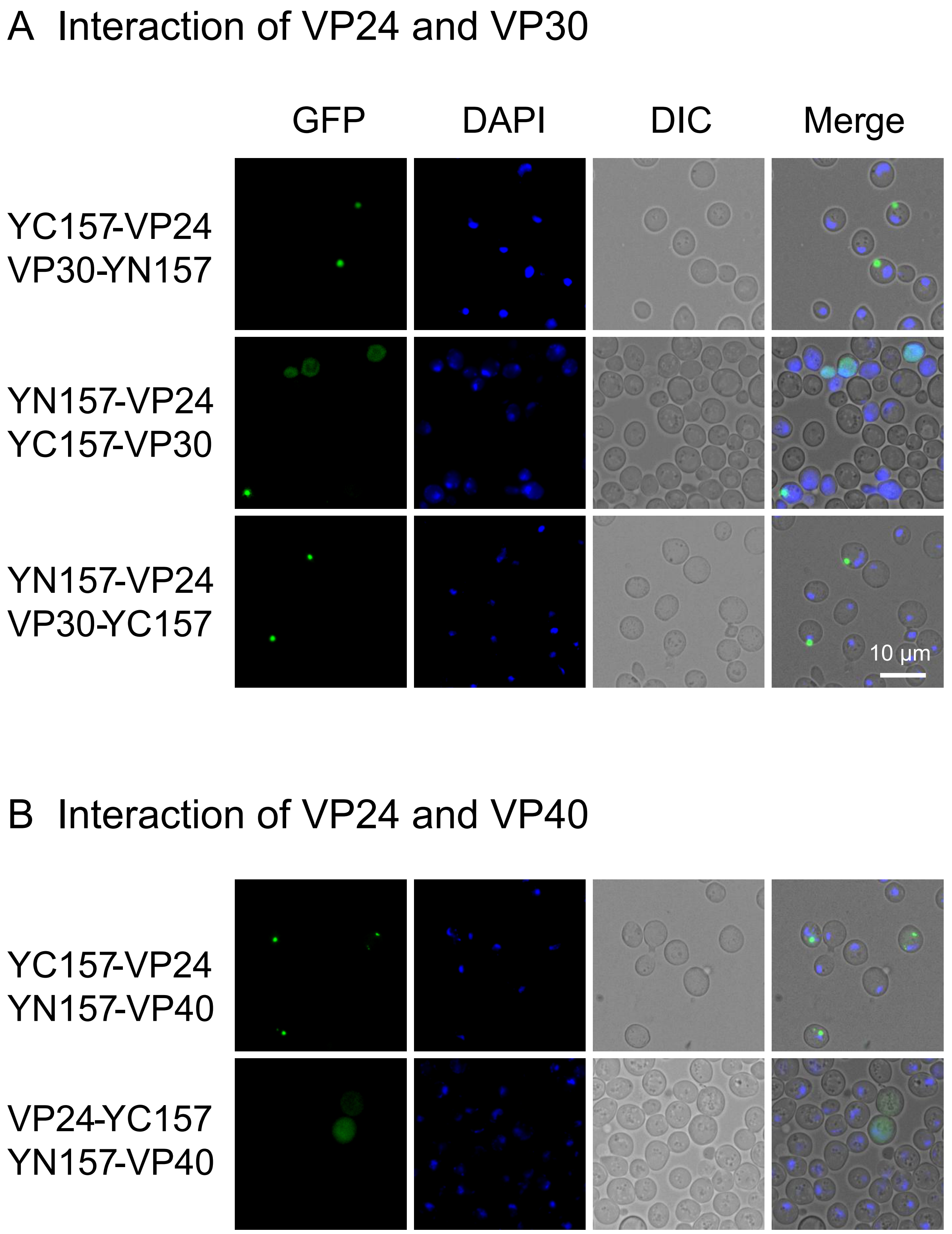
A Interaction of VP24 and VP30
GFP
DAPI
DIC
Merge
YC157-VP24
VP30-YN157
YN157-VP24
YC157-VP30
YN157-VP24
VP30-YC157
10 µm
B Interaction of VP24 and VP40
YC157-VP24
YN157-VP40
VP24-YC157
YN157-VP40

C Interaction of VP24 and NP

YN157-VP24
NP-YC157

YN157-VP24
YC157-NP

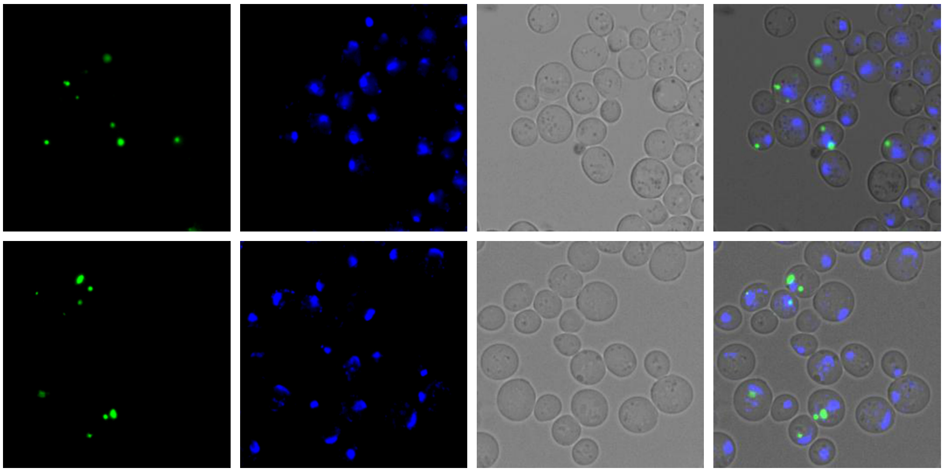

D Interaction of VP24 and GP2

GP2-YN157
VP24-YC157

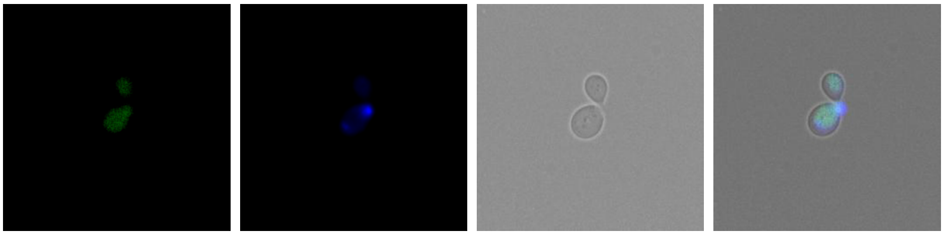

E Interaction of VP40 and VP40

YN157-VP40
YC157-VP40

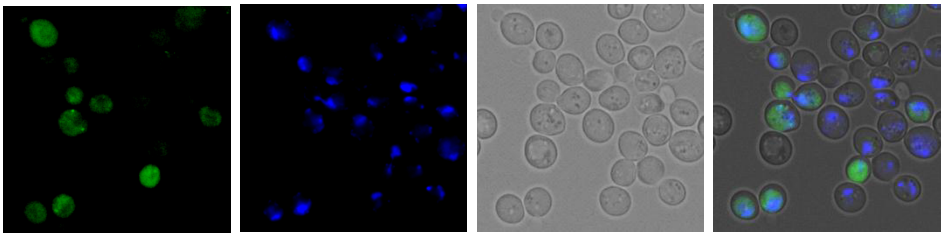

F Interaction of VP30 and VP30

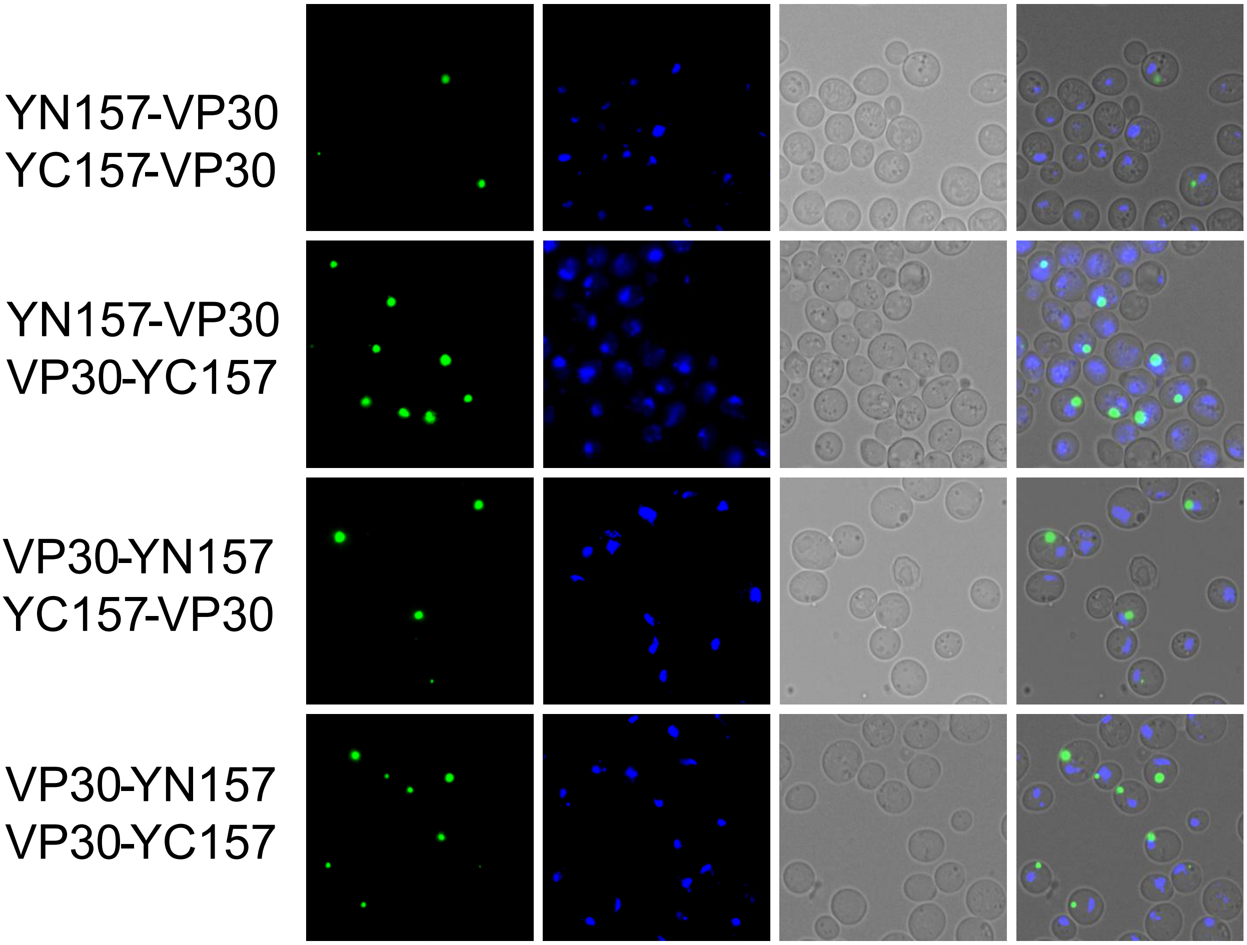

G Interaction of VP30 and VP35

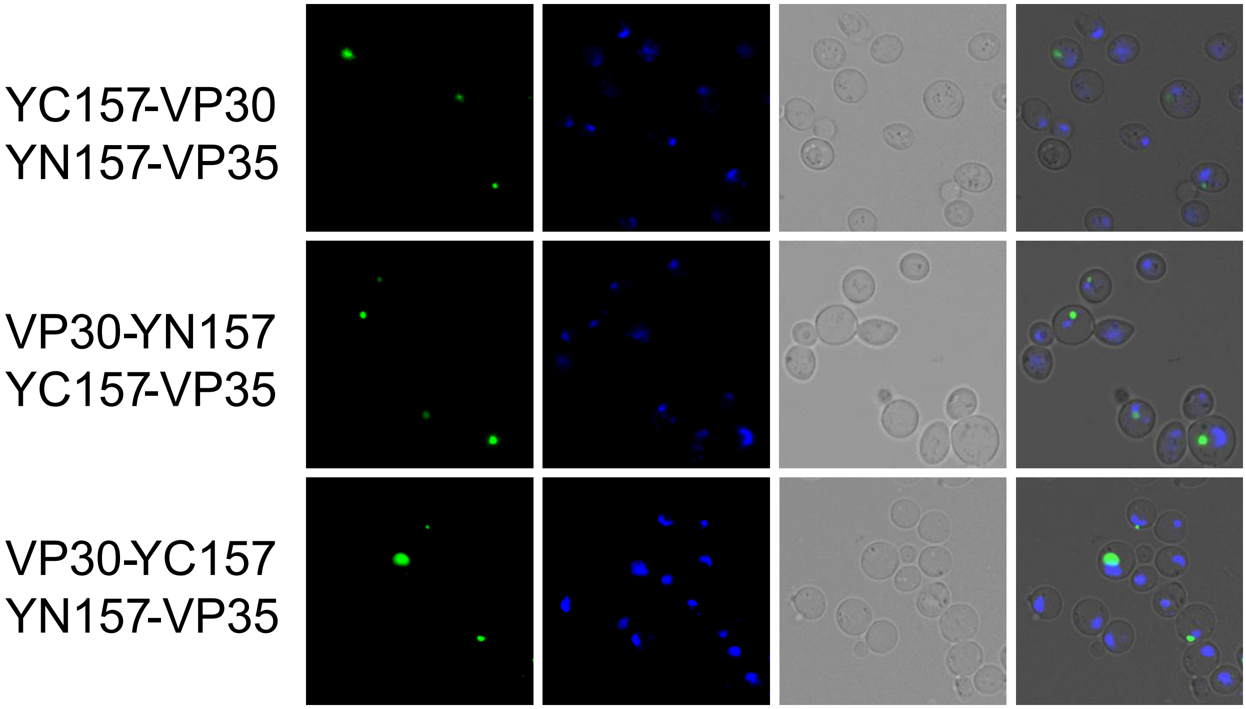

H Interaction of VP30 and VP40

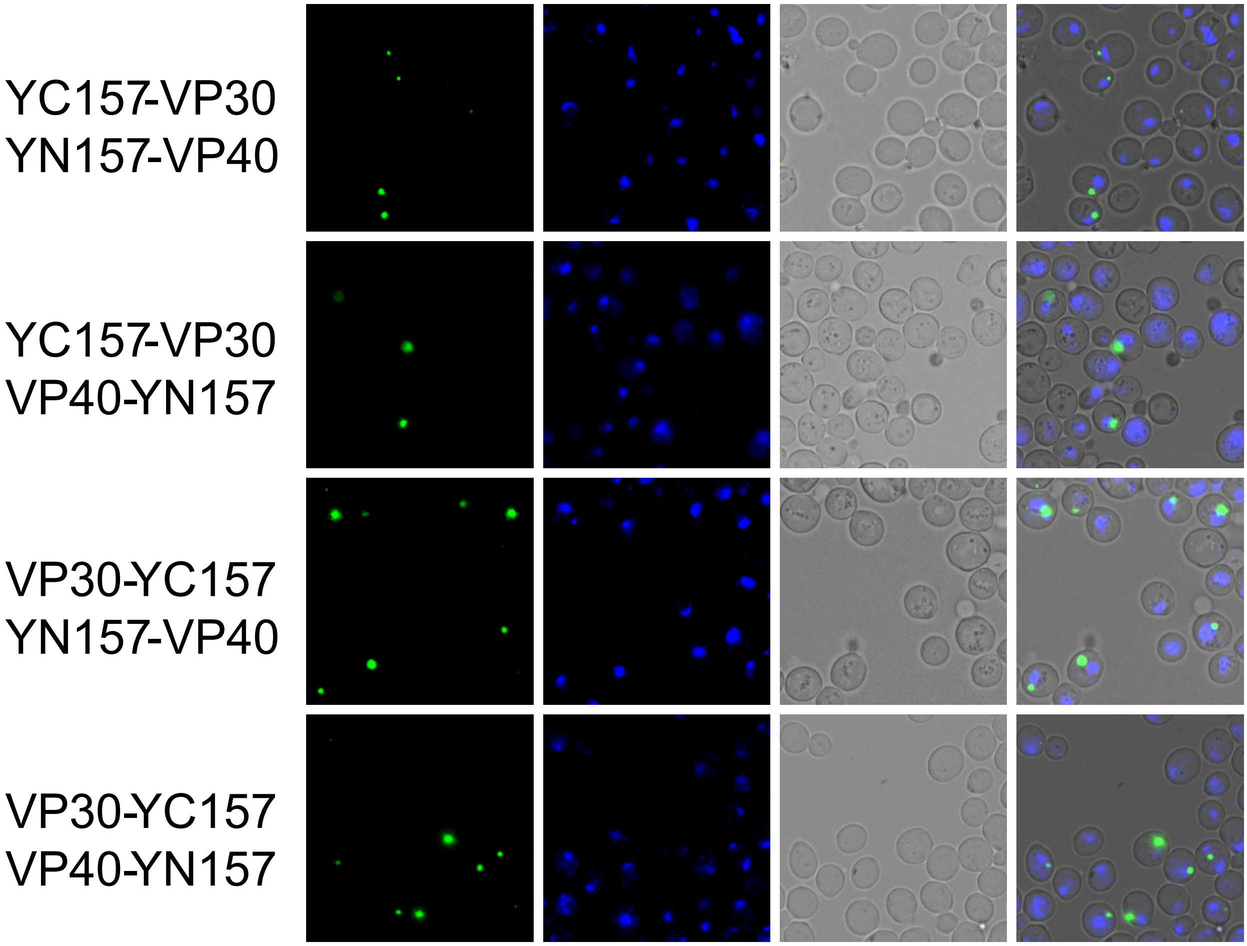

I Interaction of VP30 and NP

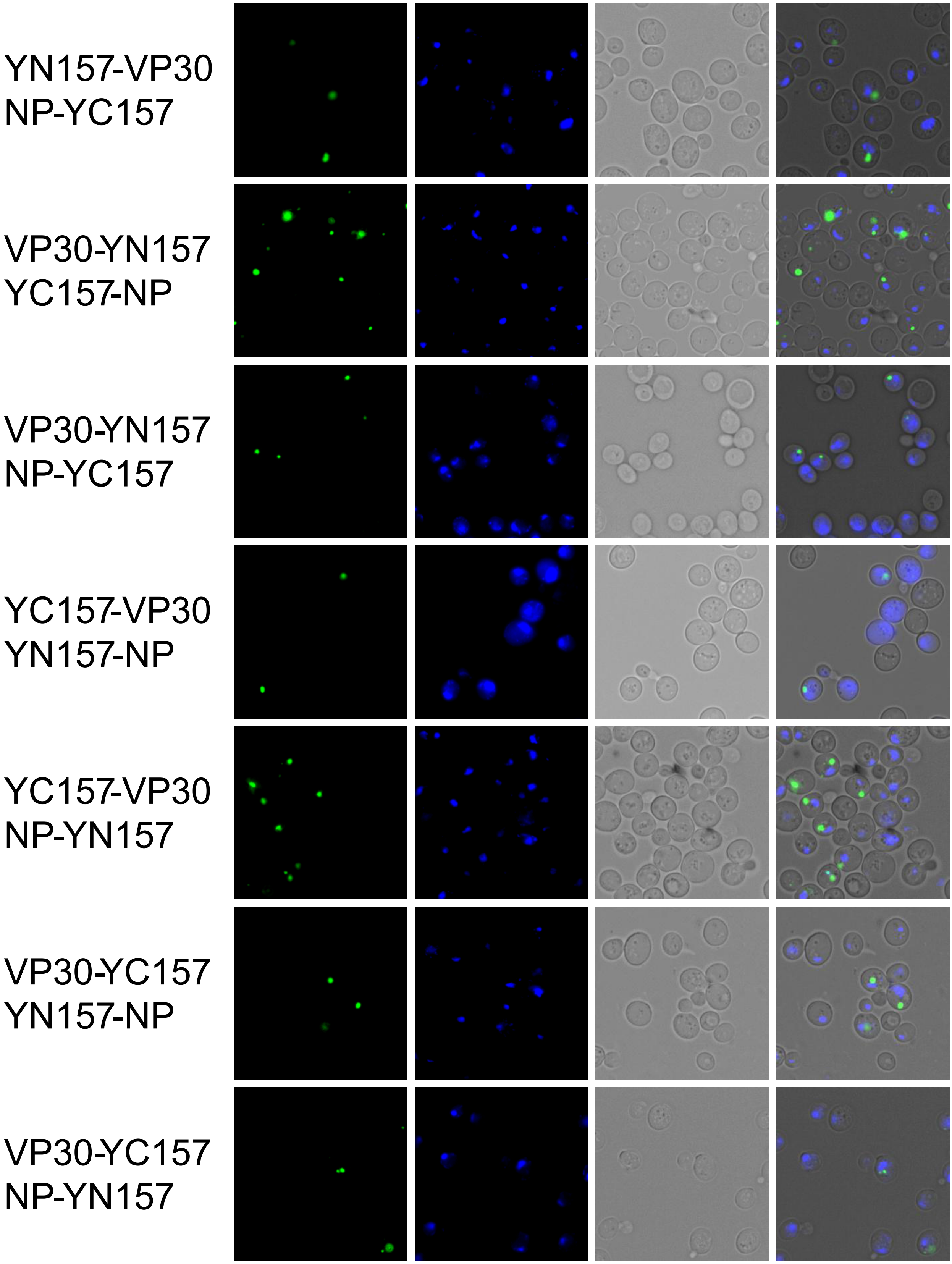

J Interaction of VP35 and VP35

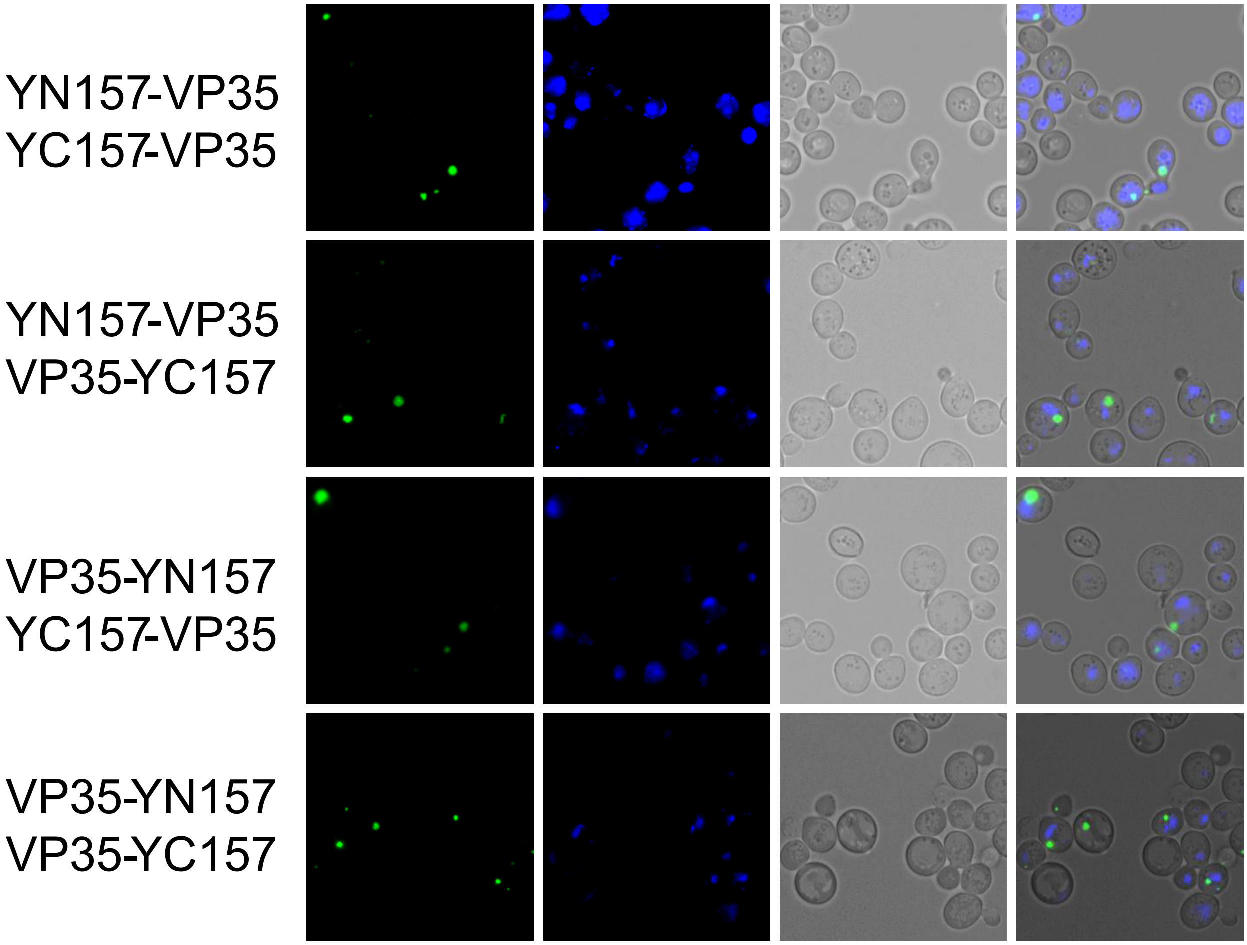

K Interaction of VP35 and VP40

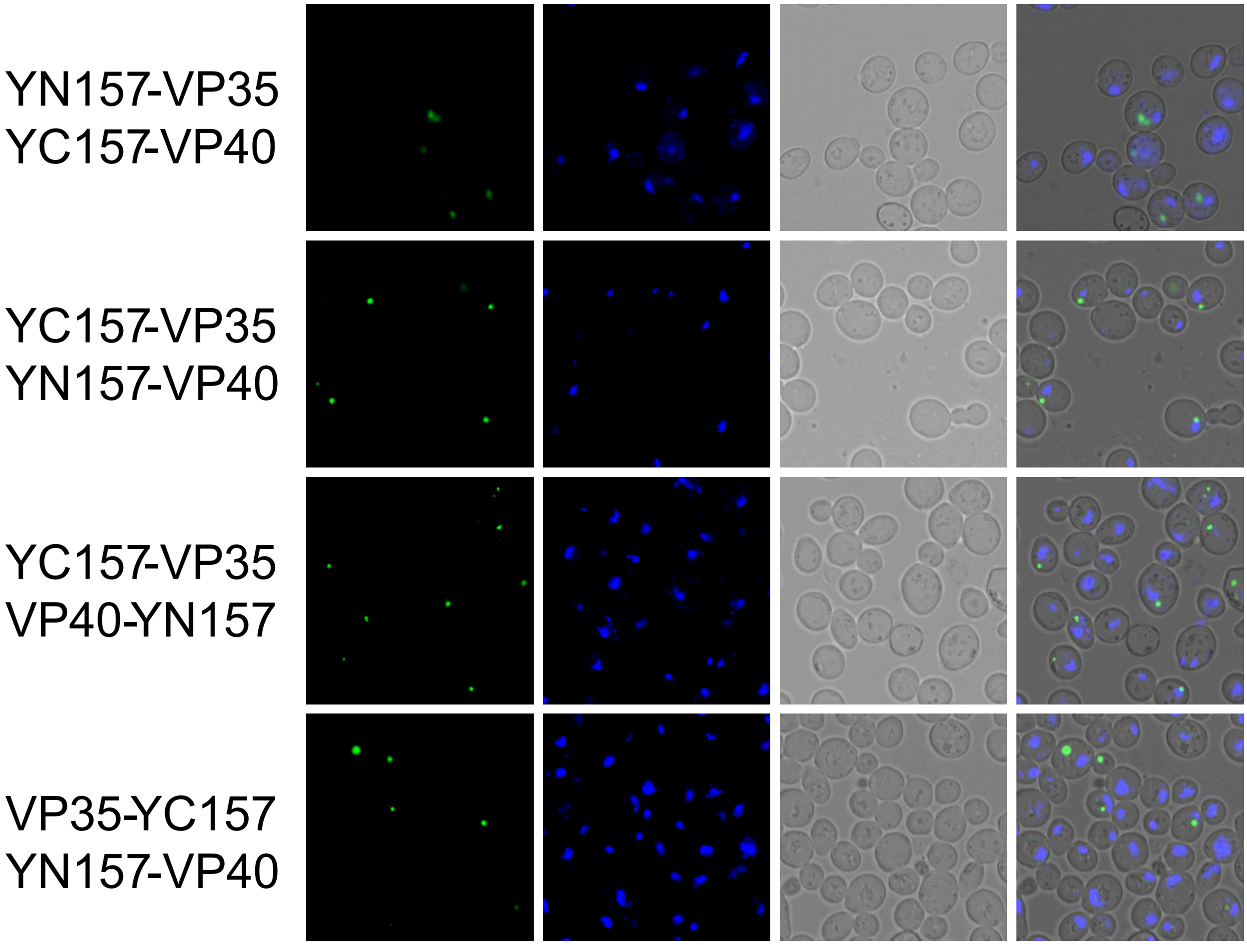

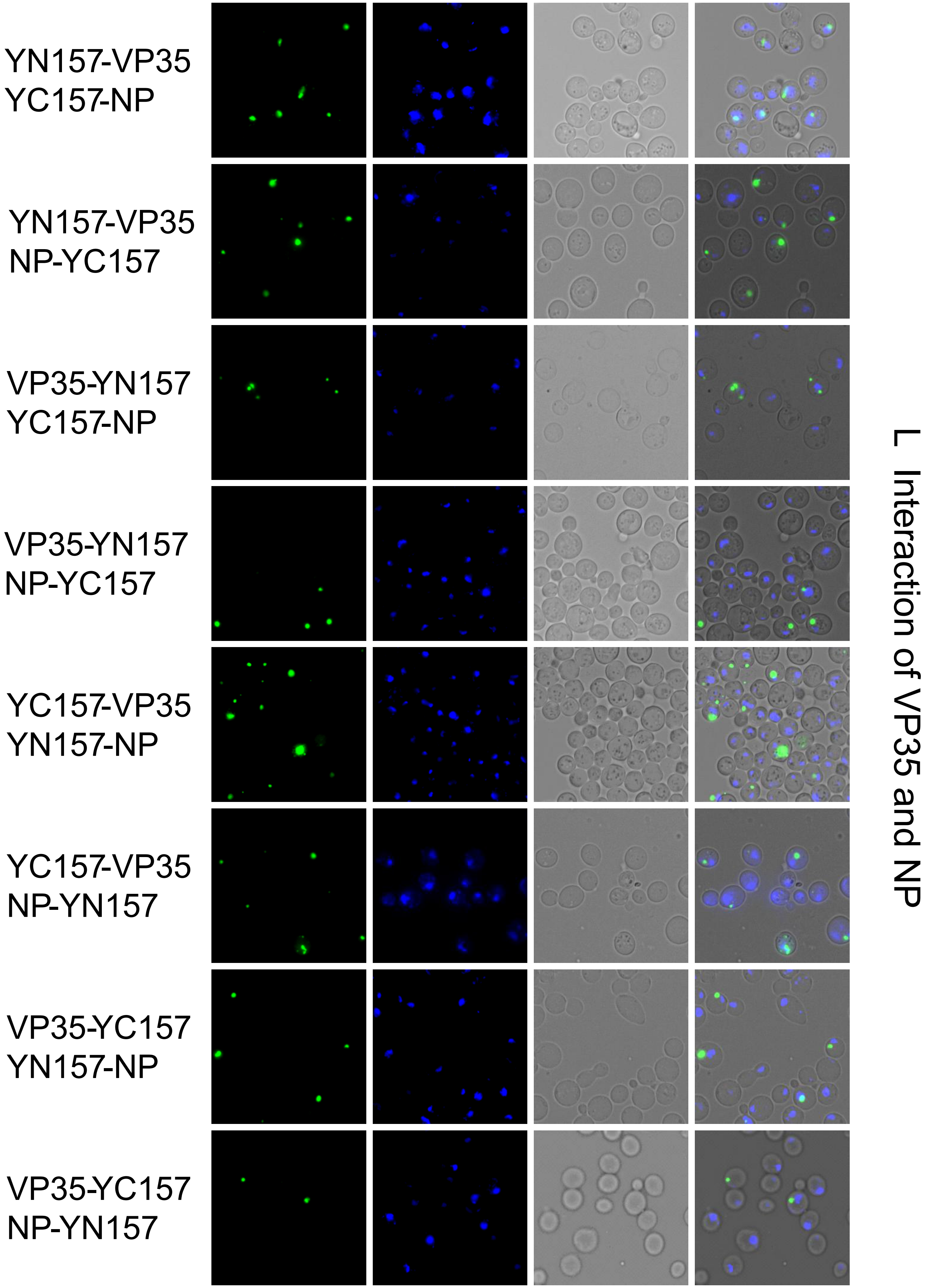
YN157-VP35
YC157-NP
YN157-VP35
NP-YC157
VP35-YN157
YC157-NP
VP35-YN157
NP-YC157
YC157-VP35
YN157-NP
YC157-VP35
NP-YN157
VP35-YC157
YN157-NP
VP35-YC157
NP-YN157
L Interaction of VP35 and NP

M Interaction of VP40 and NP

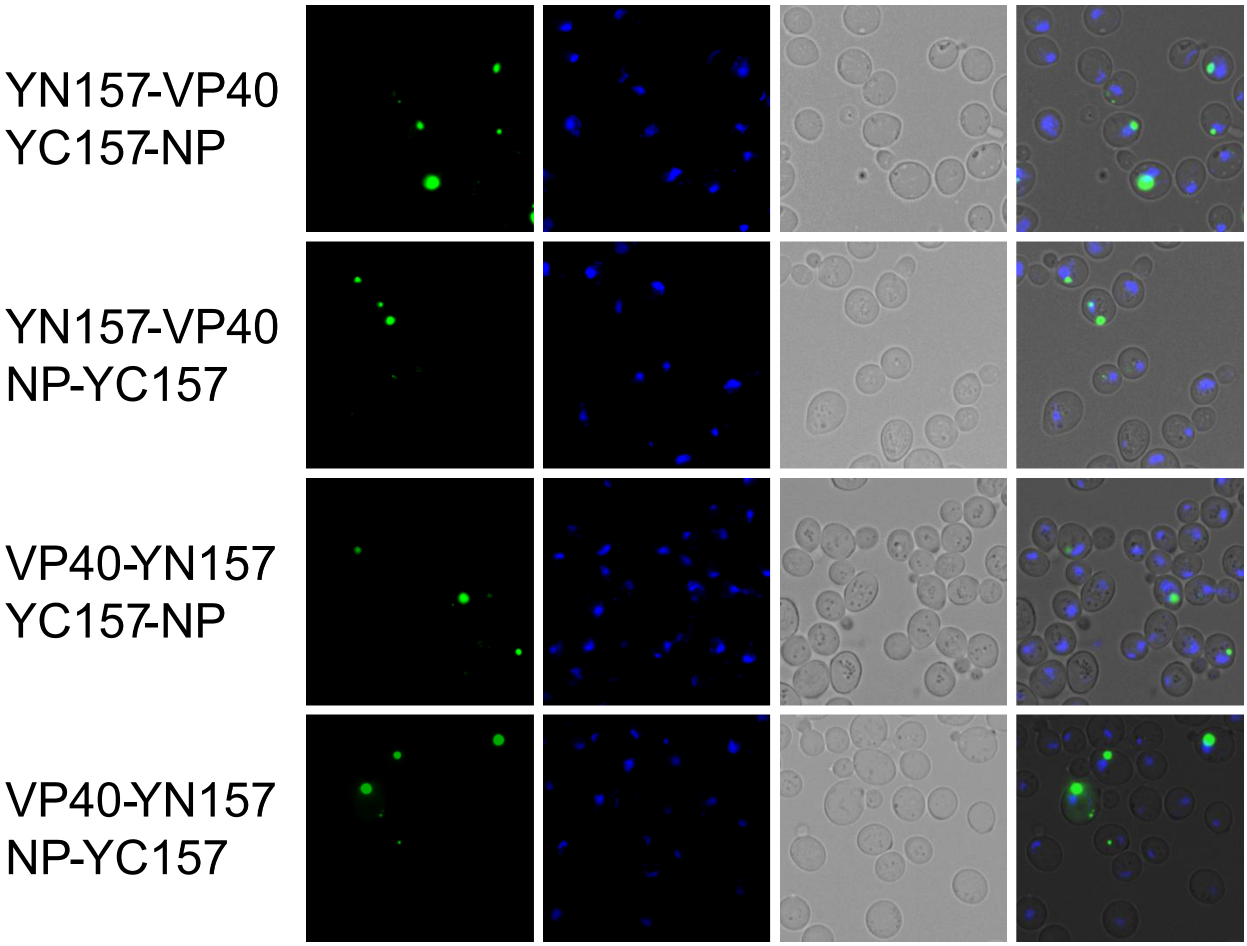

N Interaction of NP and NP

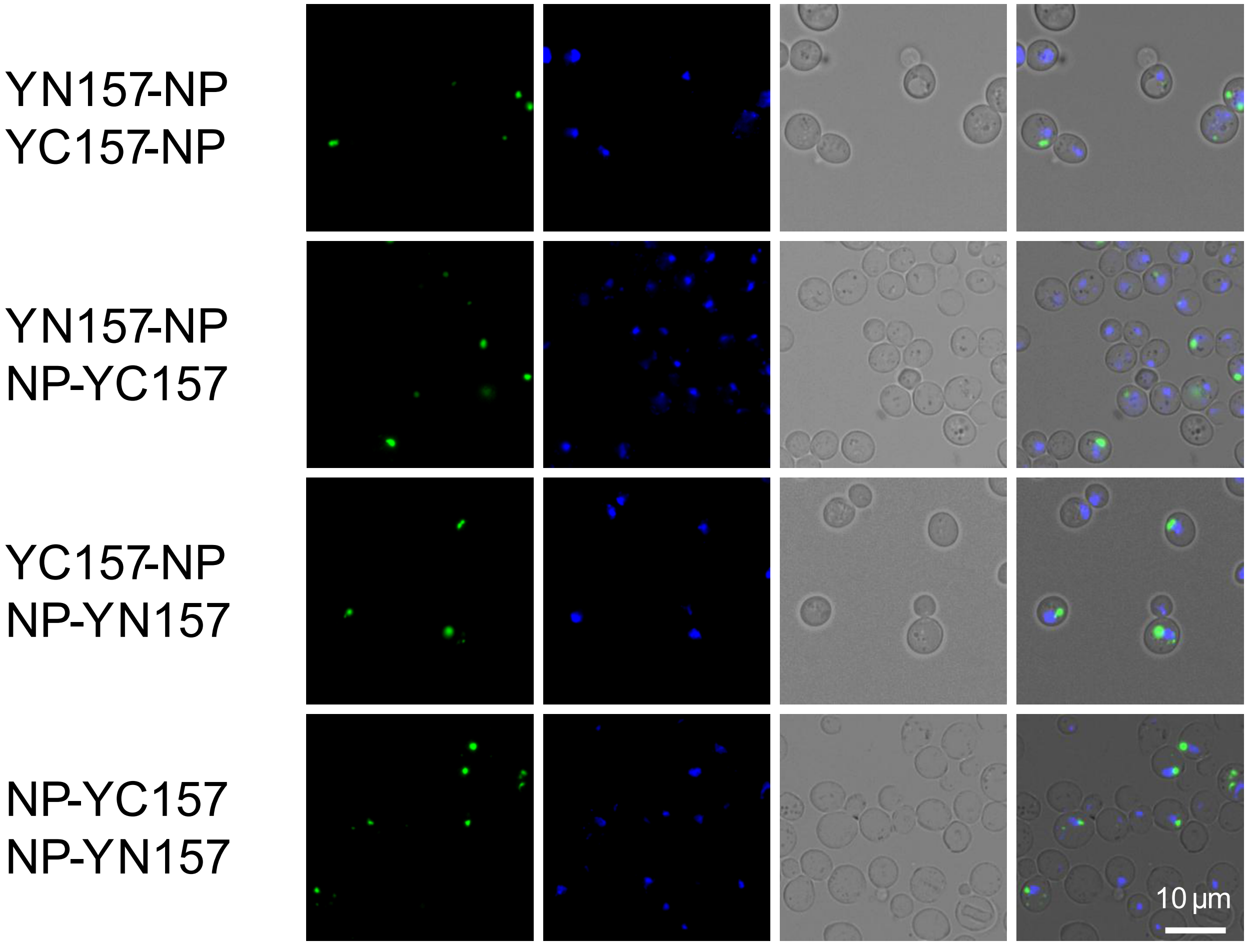

Supplement: Supplementary Figure S3 — Validation of intra EBOV PPIs by yEGFP-BiFC A.–N. To validate the intra EBOV PPIs detected by flowcytometry, EBOV proteins with yEGFP fragment tags were cotransformed into yeast cells, the fluorescence of cells was observed under the fluorescent microscope. [file mmc4.pdf]
