# Supplementary material for: A Yeast BiFC-seq Method for Genome-wide Interactome Mapping
Source: Genomics Proteomics Bioinformatics. 2021 Jul 24;20(4):795–807. doi: 10.1016/j.gpb.2021.02.008 (PMC9880813; doi:10.1016/j.gpb.2021.02.008)
Supplement: Supplementary Table S1 [file mmc12.docx]

**Table S1 The screening results of using p53 as bait by BiFC-seq**

| Group | Gene Symbol | Recorded in BioGrid Database |
| --- | --- | --- |
| YC157-p53 | AKAP1 |  |
| YC157-p53 | ALDOB |  |
| YC157-p53 | ALPL |  |
| YC157-p53 | AMZ2 |  |
| YC157-p53 | AP2M1 |  |
| YC157-p53 | ARL6IP4 |  |
| YC157-p53 | C9orf16 |  |
| YC157-p53 | CALD1 | Yes |
| YC157-p53 | CCDC74B |  |
| YC157-p53 | CCDC96 |  |
| YC157-p53 | CMTM2 |  |
| YC157-p53 | COL1A2 |  |
| YC157-p53 | CORO7 |  |
| YC157-p53 | ECHDC2 |  |
| YC157-p53 | EGFL7 |  |
| YC157-p53 | FAT2 |  |
| YC157-p53 | FTL |  |
| YC157-p53 | GSN | Yes |
| YC157-p53 | HMGN1 |  |
| YC157-p53 | HMOX1 |  |
| YC157-p53 | KANK3 |  |
| YC157-p53 | MARS |  |
| YC157-p53 | MBOAT7 |  |
| YC157-p53 | MLF2 |  |
| YC157-p53 | NDUFS6 |  |
| YC157-p53 | NDUFS8 |  |
| YC157-p53 | NSFL1C |  |
| YC157-p53 | OAZ1 |  |
| YC157-p53 | OTUB1 | Yes |
| YC157-p53 | PDZD8 |  |
| YC157-p53 | PGK1 |  |
| YC157-p53 | PLAUR |  |
| YC157-p53 | POLD2 |  |
| YC157-p53 | PREB |  |
| YC157-p53 | PRMT1 | Yes |
| YC157-p53 | PRPF3 |  |
| YC157-p53 | PSMC5 | Yes |

| Group | Gene Symbol | Recorded in BioGrid Database |
| --- | --- | --- |
| YC157-p53 | RPL18 | Yes |
| YC157-p53 | RPL29 |  |
| YC157-p53 | RPS18 | Yes |
| YC157-p53 | RPS20 |  |
| YC157-p53 | RPS3 | Yes |
| YC157-p53 | RPS5 |  |
| YC157-p53 | RRP1 | Yes |
| YC157-p53 | RUSC2 |  |
| YC157-p53 | SAMD4A |  |
| YC157-p53 | SIGMAR1 |  |
| YC157-p53 | SNRPN | Yes |
| YC157-p53 | TAGLN |  |
| YC157-p53 | TMSB4X | Yes |
| YC157-p53 | TOMM7 |  |
| YC157-p53 | TSC2 |  |
| YC157-p53 | ZDHHC12 |  |
| YC157-p53 | ZNF365 |  |
| p53-YC157 | ABCD4 |  |
| p53-YC157 | C16orf13 |  |
| p53-YC157 | CAMLG |  |
| p53-YC157 | CCDC159 |  |
| p53-YC157 | CCT3 | Yes |
| p53-YC157 | CD63 |  |
| p53-YC157 | CDK5 | Yes |
| p53-YC157 | CPSF4L |  |
| p53-YC157 | CRIP2 |  |
| p53-YC157 | CRYAB | Yes |
| p53-YC157 | DTNBP1 |  |
| p53-YC157 | FBXW5 |  |
| p53-YC157 | GTF3C6 |  |
| p53-YC157 | GZF1 |  |
| p53-YC157 | HLA-DMB |  |
| p53-YC157 | IFI6 |  |
| p53-YC157 | IFITM3 |  |
| p53-YC157 | IGLL1 |  |
| p53-YC157 | JUP |  |
| p53-YC157 | KLHDC9 |  |
| p53-YC157 | MPC2 |  |
| p53-YC157 | MPLKIP |  |
| p53-YC157 | NCAPH2 |  |
| p53-YC157 | NDN | Yes |
| p53-YC157 | NECAB2 |  |

| Group | Gene Symbol | Recorded in BioGrid Database |
| --- | --- | --- |
| p53-YC157 | NEDD9 |  |
| p53-YC157 | NFKBIA | Yes |
| p53-YC157 | PDZK1IP1 |  |
| p53-YC157 | RPL28 | Yes |
| p53-YC157 | RPL7A | Yes |
| p53-YC157 | SCAMP3 |  |
| p53-YC157 | SLIT3 |  |
| p53-YC157 | SUPT7L | Yes |
| p53-YC157 | TCTN3 |  |
| p53-YC157 | TXNDC2 |  |
| p53-YC157 | YWHAZ | Yes |
| p53 | ARRDC5 |  |
| p53 | EIF4G2 |  |
| p53 | MSRA |  |
| p53 | PPM1G |  |
| p53 | RSRP1 |  |
| p53 | SPATS2 |  |
| p53 | TPI1 | Yes |

*Note*: Gene Symbol, NCBI ENTREZ Symbols of the interactors; Group, Specific highly expressed transcripts in each group according to the different expression analysis. Here, p53 indicates the overlap interactors using p53-YC157 and YC157-p53 as bait.
