# Supplementary material for: A Yeast BiFC-seq Method for Genome-wide Interactome Mapping
Source: Genomics Proteomics Bioinformatics. 2021 Jul 24;20(4):795–807. doi: 10.1016/j.gpb.2021.02.008 (PMC9880813; doi:10.1016/j.gpb.2021.02.008)
Supplement: Supplementary Table S2 [file mmc13.docx]

**Table S2 Evaluation of the reliability of the p53 interactions by PRINCESS**

| Evidence | Interactions submitted | High confidence, LR>2.0 | PPI database | Interolog | Interacting domain | GO coannotation | Genome context | Gene coexpression | Network topology |
| --- | --- | --- | --- | --- | --- | --- | --- | --- | --- |
| #interaction | 53 | 34 | 0 | 0 | 8 | 34 | 0 | 11 | 10 |

*Note*: Confidence scores were measured by a Bayesian approach that combinesbiological evidence from multiple sources. #interaction, the number of interactions in each category; LR, likelihood ratio.
