# Supplementary material for: A Yeast BiFC-seq Method for Genome-wide Interactome Mapping
Source: Genomics Proteomics Bioinformatics. 2021 Jul 24;20(4):795–807. doi: 10.1016/j.gpb.2021.02.008 (PMC9880813; doi:10.1016/j.gpb.2021.02.008)
Supplement: Supplementary Table S7 [file mmc18.docx]

**Table S7 A genome-wide BiFC-seq PPIs screening results**

| Gene Symbol_a | Gene Symbol_b | Fluorescence intensity | Recorded in BioGrid Database |
| --- | --- | --- | --- |
| RPL30 | RPL13 | high | Yes |
| RPL9 | RPL27A | high | Yes |
| VIMP | UFD1L | high | Yes |
| HLA-A | GAP43 | high |  |
| ANKMY2 | TPT1 | high |  |
| ANKMY2 | APOE | high |  |
| ANKMY2 | RPL29 | high |  |
| BRD3 | TMBIM6 | high |  |
| BRD3 | ALDOA | high |  |
| C17orf70 | PABPC1 | high |  |
| C4orf3 | CCDC89 | high |  |
| C4orf3 | MYLK | high |  |
| C4orf3 | HADHA | high |  |
| C4orf3 | MGST1 | high |  |
| C4orf3 | SHARPIN | high |  |
| C4orf3 | MT3 | high |  |
| C4orf3 | ARL3 | high |  |
| C4orf3 | GAP43 | high |  |
| CD81 | PDHA2 | high |  |
| CD81 | BFSP1 | high |  |
| CERS2 | ALDOA | high |  |
| CMTM2 | TBCB | high |  |
| CMTM2 | UBE2S | high |  |
| CMTM2 | TPM2 | high |  |
| CREG1 | TMSB4X | high |  |
| CST3 | RPL23A | high |  |
| DAXX | ANXA2 | high |  |
| EIF1 | APRT | high |  |
| EIF1 | EEF1G | high |  |
| FBXW5 | BFSP1 | high |  |
| FRG1 | PABPC1 | high |  |

| Gene Symbol_a | Gene Symbol_b | | Fluorescence intensity | | | | Recorded in BioGrid  Database | | |  |
| --- | --- | --- | --- | --- | --- | --- | --- | --- | --- | --- |
| FXYD2 | CCDC89 | | high | | | |  | | |  |
| GPX4 | RBM34 | | high | | | |  | | |  |
| GTPBP6 | ARHGAP42 | | high | | | |  | | |  |
| GTPBP6 | EEF1G | | high | | | |  | | |  |
| GTPBP6 | RPL31 | | high | | | |  | | |  |
| GTPBP6 | UFD1L | | high | | | |  | | |  |
| H3F3B | SPATA7 | | high | | | |  | | |  |
| HMGCL | UFD1L | | high | | | |  | | |  |
| HMGCL | PABPC1 | | high | | | |  | | |  |
| KAT8 | CCL2 | | high | | | |  | | |  |
| MBP | EEF1G | | high | | | |  | | |  |
| MT1F | PSMC3 | | high | | | |  | | |  |
| MT1F | TMEM223 | | high | | | |  | | |  |
| NME2 | TMBIM6 | | high | | | |  | | |  |
| NPIPB5 | AKR7A3 | | high | | | |  | | |  |
| NPIPB5 | PRM2 | | high | | | |  | | |  |
| OTUB1 | TSSC4 | | high | | | |  | | |  |
| OTUB1 | ATXN2L | | high | | | |  | | |  |
| OTUB1 | REPIN1 | | high | | | |  | | |  |
| OTUB1 | EEF1G | | high | | | |  | | |  |
| OTUB1 | CCDC89 | | high | | | |  | | |  |
| PDZK1IP1 | GTF3C1 | | high | | | |  | | |  |
| PDZK1IP1 | HADHA | | high | | | |  | | |  |
| PFDN5 | APOE | | high | | | |  | | |  |
| PFDN5 | VAMP2 | | high | | | |  | | |  |
| PFDN5 | RPL13 | | high | | | |  | | |  |
| PFDN5 | ALDOB | | high | | | |  | | |  |
| PFDN5 | RPL31 | | high | | | |  | | |  |
| PNPLA6 | PCBP1 | | high | | | |  | | |  |
| PRM2 | CCDC89 | | high | | | |  | | |  |
| PRM3 | HLA-C | | high | | | |  | | |  |
| PRPF6 | TMSB10 | | high | | | |  | | |  |
| PSMD8 | RPL31 | | high | | | |  | | |  |
| RBP1 | CD99 | | high | | | |  | | |  |
| RBP1 | PEBP1 | | high | | | |  | | |  |
| RBP1 | PABPC1 | | high | | | |  | | |  |
| RPL23A | VDAC1 | | high | | | |  | | |  |
| RPL23A | CSDE1 | | high | | | |  | | |  |
| RPL23A | LRRC48 | | high | | | |  | | |  |
| RPL29 | ARL3 | | high | | | |  | | |  |
| RPL29 | DHFR | | high | | | |  | | |  |
| RPL8 | REPIN1 | | high | | | |  | | |  |
| Gene Symbol_a | | Gene Symbol_b | | | Fluorescence intensity | | | Recorded in BioGrid Database | |  |
| RPS11 | | CST3 | | | high | | |  |  |  |
| RPS19 | | CCDC89 | | | high | | |  |  |  |
| RPS19 | | PUF60 | | | high | | |  |  |  |
| RPS20 | | CIRBP | | | high | | |  |  |  |
| S100A9 | | APOE | | | high | | |  |  |  |
| SDSL | | UFD1L | | | high | | |  |  |  |
| SFTPC | | AKR7A3 | | | high | | |  |  |  |
| SLCO3A1 | | MIOX | | | high | | |  |  |  |
| STMN1 | | CCDC89 | | | high | | |  |  |  |
| TIGD1 | | MGST1 | | | high | | |  |  |  |
| TIGD1 | | MYLK | | | high | | |  |  |  |
| TIGD1 | | APOE | | | high | | |  |  |  |
| TPM2 | | KRT18 | | | high | | |  |  |  |
| TREX1 | | TMEM109 | | | high | | |  |  |  |
| TRIM28 | | ARL3 | | | high | | |  |  |  |
| TSPAN16 | | MT1G | | | high | | |  |  |  |
| TXNDC2 | | PSMD10 | | | high | | |  |  |  |
| UBIAD1 | | ARHGAP42 | | | high | | |  |  |  |
| UBIAD1 | | MT3 | | | high | | |  |  |  |
| VIMP | | CCDC89 | | | high | | |  |  |  |
| VIMP | | OBSL1 | | | high | | |  |  |  |
| VIMP | | RPL23A | | | high | | |  |  |  |
| VIMP | | NHP2L1 | | | high | | |  |  |  |
| VIMP | | CCL2 | | | high | | |  |  |  |
| VIMP | | ALDOA | | | high | | |  |  |  |
| VWF | | SPATA7 | | | high | | |  |  |  |
| ZNF365 | | BFSP1 | | | high | | |  |  |  |
| HMGN1 | | AKR1C3 | | | high | | |  |  |  |
| PDZK1IP1 | | RPL11 | | | high | | |  |  |  |
| YBX1 | | C1QTNF2 | | | high | | |  |  |  |
| YBX1 | | RPL31 | | | high | | |  |  |  |
| ANAPC13 | | EEF1G | | | high | | |  |  |  |
| ANXA6 | | PGAM2 | | | high | | |  |  |  |
| RPL30 | | EEF1G | | | high | | |  |  |  |
| ANXA6 | | RPL31 | | | high | | |  |  |  |
| FXYD2 | | ALDOA | | | high | | |  |  |  |
| RPS13 | | PGS1 | | | high | | |  |  |  |
| RPS11 | | PTGES3L-AARSD1 | | | high | | |  |  |  |
| SFTPC | | CALM3 | | | high | | |  |  |  |
| OTUB1 | | PRDX1 | | | medium | | | Yes |  |  |
| RPL30 | | RPS5 | | | medium | | | Yes |  |  |
| ANKMY2 | | SH3RF2 | | | medium | | |  |  |  |
| Gene Symbol_a | Gene Symbol_b | | | Fluorescence intensity | | | Recorded in BioGrid  Database | | | |
| ANKMY2 | TPT1 | | | medium | | |  | | | |
| APOE | EEF1G | | | medium | | |  | | | |
| APOE | EIF1 | | | medium | | |  | | | |
| ARL2 | MT1G | | | medium | | |  | | | |
| ATP1A1 | TBRG4 | | | medium | | |  | | | |
| B3GALT6 | GSTO1 | | | medium | | |  | | | |
| BRD3 | TMBIM6 | | | medium | | |  | | | |
| BRD3 | CCDC89 | | | medium | | |  | | | |
| CD74 | ATP6AP1 | | | medium | | |  | | | |
| CERS2 | RPL35 | | | medium | | |  | | | |
| CERS2 | RPL30 | | | medium | | |  | | | |
| COX8A | RPL11 | | | medium | | |  | | | |
| COX8A | PSMC3 | | | medium | | |  | | | |
| DHRS4 | SLC25A3 | | | medium | | |  | | | |
| EEF1A1 | AARSD1 | | | medium | | |  | | | |
| FCGRT | TMSB4X | | | medium | | |  | | | |
| FXYD2 | RPL31 | | | medium | | |  | | | |
| HMGCL | UFD1L | | | medium | | |  | | | |
| HMGN1 | SORD | | | medium | | |  | | | |
| KRT19 | PRDX5 | | | medium | | |  | | | |
| MBP | EEF1G | | | medium | | |  | | | |
| MBP | RPL31 | | | medium | | |  | | | |
| MORN3 | TMSB4X | | | medium | | |  | | | |
| MT1F | TMEM223 | | | medium | | |  | | | |
| MVD | TARBP2 | | | medium | | |  | | | |
| OTUB1 | TSSC4 | | | medium | | |  | | | |
| PCK1 | CSTA | | | medium | | |  | | | |
| PNPLA6 | PCBP1 | | | medium | | |  | | | |
| PRM2 | CCDC89 | | | medium | | |  | | | |
| PRR13 | TMBIM6 | | | medium | | |  | | | |
| PSAP | LAD1 | | | medium | | |  | | | |
| PSMB6 | SNRPB2 | | | medium | | |  | | | |
| RHPN1 | CCS | | | medium | | |  | | | |
| RPL19 | PSMC3 | | | medium | | |  | | | |
| RPL23A | ATP5G2 | | | medium | | |  | | | |
| RPL23A | COX6B1 | | | medium | | |  | | | |
| RPL30 | CCDC89 | | | medium | | |  | | | |
| RPL30 | CHKB | | | medium | | |  | | | |
| RPL7A | EEF1G | | | medium | | |  | | | |
| RPL9 | EEF1G | | | medium | | |  | | | |
| RPS19 | PUF60 | | | medium | | |  | | | |
| SMG6 | CCZ1B | | | medium | | |  | | | |
| Gene Symbol_a | Gene Symbol_b | | Fluorescence intensity | | | Recorded in BioGrid  Database | | | |  |
| TREX1 | TMEM109 | | medium | | |  | | | |  |
| VIMP | RPS3A | | medium | | |  | | | |  |
| VWA9 | DCXR | | medium | | |  | | | |  |
| RPL23A | RPL31 | | low | | | Yes | | | |  |
| RPL23A | GOSR1 | | low | | | Yes | | | |  |
| RPL23A | RPS3A | | low | | | Yes | | | |  |
| RPL9 | RPL31 | | low | | | Yes | | | |  |
| RPS11 | RPL31 | | low | | | Yes | | | |  |
| RPS13 | RPL31 | | low | | | Yes | | | |  |
| RPL13 | RPL31 | | low | | | Yes | | | |  |
| ALDH1A1 | RPL31 | | low | | |  | | | |  |
| ALKBH7 | RPL31 | | low | | |  | | | |  |
| APOE | RPL31 | | low | | |  | | | |  |
| APOE | HOPX | | low | | |  | | | |  |
| APOE | ARL3 | | low | | |  | | | |  |
| ARL2 | MT1G | | low | | |  | | | |  |
| ARL2 | RPL31 | | low | | |  | | | |  |
| BRD3 | TMBIM6 | | low | | |  | | | |  |
| EEFSEC | SLC27A6 | | low | | |  | | | |  |
| EEFSEC | PTGES3L-AARSD1 | | low | | |  | | | |  |
| EIF4A1 | TPT1 | | low | | |  | | | |  |
| FKBP6 | HOPX | | low | | |  | | | |  |
| FXYD2 | RPL31 | | low | | |  | | | |  |
| FXYD2 | WDR59 | | low | | |  | | | |  |
| FXYD2 | PTGES3L-AARSD1 | | low | | |  | | | |  |
| FXYD2 | RPL14 | | low | | |  | | | |  |
| FXYD2 | MST1L | | low | | |  | | | |  |
| FXYD2 | ARL3 | | low | | |  | | | |  |
| FXYD2 | RPL26 | | low | | |  | | | |  |
| FXYD2 | HOPX | | low | | |  | | | |  |
| FXYD2 | GSTP1 | | low | | |  | | | |  |
| FXYD2 | PIN1 | | low | | |  | | | |  |
| FXYD2 | EEF1G | | low | | |  | | | |  |
| FXYD2 | MT1G | | low | | |  | | | |  |
| FXYD2 | PABPC1 | | low | | |  | | | |  |
| FXYD2 | RPL35 | | low | | |  | | | |  |
| FXYD2 | AARSD1 | | low | | |  | | | |  |
| FXYD2 | CCDC89 | | low | | |  | | | |  |
| FXYD2 | RPL9 | | low | | |  | | | |  |
| FXYD2 | TMBIM6 | | low | | |  | | | |  |
| FXYD2 | MIOX | | low | | |  | | | |  |
| FXYD2 | SLC27A6 | | low | | |  | | | |  |
| Gene Symbol_a | Gene Symbol_b | | Fluorescence intensity | | | | Recorded in BioGrid  Database | | |  |
| FXYD2 | UBE2V2 | | low | | | |  | | |  |
| FXYD2 | TMSB4X | | low | | | |  | | |  |
| FXYD2 | EIF1 | | low | | | |  | | |  |
| FXYD2 | CSTA | | low | | | |  | | |  |
| HMGN1 | MT3 | | low | | | |  | | |  |
| KNDC1 | ARL3 | | low | | | |  | | |  |
| MAP2 | RPL31 | | low | | | |  | | |  |
| MCM7 | GOSR1 | | low | | | |  | | |  |
| MPC2 | RPL31 | | low | | | |  | | |  |
| MPC2 | GOSR1 | | low | | | |  | | |  |
| MT1F | MST1L | | low | | | |  | | |  |
| NDRG2 | HNRNPC | | low | | | |  | | |  |
| NDRG2 | RPL31 | | low | | | |  | | |  |
| NDRG2 | S100A6 | | low | | | |  | | |  |
| PCK1 | CSTA | | low | | | |  | | |  |
| PDXK | TMSB4X | | low | | | |  | | |  |
| PDZK1IP1 | RPL11 | | low | | | |  | | |  |
| PDZK1IP1 | ARL3 | | low | | | |  | | |  |
| PDZK1IP1 | RPL31 | | low | | | |  | | |  |
| PFDN5 | APOE | | low | | | |  | | |  |
| PFDN5 | C11orf58 | | low | | | |  | | |  |
| PPM1G | HOPX | | low | | | |  | | |  |
| PSMD3 | TMSB4X | | low | | | |  | | |  |
| PTGES3L-AARSD1 | RPL31 | | low | | | |  | | |  |
| RABAC1 | GSTP1 | | low | | | |  | | |  |
| RABAC1 | AASDHPPT | | low | | | |  | | |  |
| RPL10 | MT3 | | low | | | |  | | |  |
| RPL23A | AASDHPPT | | low | | | |  | | |  |
| RPL23A | ARL3 | | low | | | |  | | |  |
| RPL23A | HOPX | | low | | | |  | | |  |
| RPL23A | EXOSC5 | | low | | | |  | | |  |
| RPL23A | RPL26 | | low | | | |  | | |  |
| RPL23A | COX6B1 | | low | | | |  | | |  |
| RPL23A | CLU | | low | | | |  | | |  |
| RPS11 | PTGES3L-AARSD1 | | low | | | |  | | |  |
| RPS11 | PIN1 | | low | | | |  | | |  |
| RPS11 | SLC27A6 | | low | | | |  | | |  |
| RPS11 | IAH1 | | low | | | |  | | |  |
| RPS11 | HOPX | | low | | | |  | | |  |
| RPS11 | MT3 | | low | | | |  | | |  |
| RPS11 | LOC81691 | | low | | | |  | | |  |
| RPS11 | AASDHPPT | | low | | | |  | | |  |

| Gene Symbol_a | Gene Symbol_b | Fluorescence intensity | | Recorded in BioGrid  Database |
| --- | --- | --- | --- | --- |
| RPS11 | ANKRD39 | low |  | |
| RPS13 | EIF1 | low |  | |
| RPS4Y1 | CSTA | low |  | |
| SAFB2 | RPL31 | low |  | |
| SMARCA2 | PTGES3L-AARSD1 | low |  | |
| SNX12 | RPL31 | low |  | |
| TSACC | TPT1 | low |  | |

*Note*: The reads of the same amplicon derived from YN157-library and YC157-library were mapped to the genome CDS regions individually. The gene symbols of the interactors from three screening groups were shown.
