# Supplementary material for: A Yeast BiFC-seq Method for Genome-wide Interactome Mapping
Source: Genomics Proteomics Bioinformatics. 2021 Jul 24;20(4):795–807. doi: 10.1016/j.gpb.2021.02.008 (PMC9880813; doi:10.1016/j.gpb.2021.02.008)
Supplement: Supplementary Table S8 [file mmc19.docx]

**Table S8 Evaluation of the reliability of the genome-wide PPI screening results by PRINCESS**

| Evidence | Interactions Submitted | High Confidence, LR>2.0 | PPI Database | Interolog | Interacting Domain | GO Coannotation | Genome Context | Gene Coexpression | Network Topology |
| --- | --- | --- | --- | --- | --- | --- | --- | --- | --- |
| #interaction | 137 | 26 | 0 | 0 | 14 | 37 | 0 | 19 | 0 |

*Note*: Confidence scores were measured by a Bayesian approach that combines biological evidence from multiple sources. #interaction, the number of interactions in each category; LR, likelihood ratio; PPI, protein-protein interaction; GO, gene ontology.
