# Supplementary material for: A Yeast BiFC-seq Method for Genome-wide Interactome Mapping
Source: Genomics Proteomics Bioinformatics. 2021 Jul 24;20(4):795–807. doi: 10.1016/j.gpb.2021.02.008 (PMC9880813; doi:10.1016/j.gpb.2021.02.008)
Supplement: Supplementary Table S9 [file mmc20.docx]

**Table S9 Co-expression analysis of PPI pairs identified by BiFC-seq**

|  | No. of PPIs | Co-expression  (SCC > 0.5) | Ratio of  co-expression |
| --- | --- | --- | --- |
| Negatome | 1152 | 131 | 0.113715 |
| p53 interactors | 96 | 20 | 0.208333 |
| Genome-wide interactors | 214 | 28 | 0.130841 |

*Note*: BiFC-seq identified PPI pairs were compared with human negatome dataset, which is a collection of protein and domain pairs that are unlikely engaged in direct physical interactions, these with absolute value of spearman correlation coefficient (SCC)>0.5 were considered co-expression pairs. PPI, protein-protein interaction.
